# Peptidomimetics Activating the Proteasome: A New Perspective for Parkinson’s Treatment

**DOI:** 10.1021/acs.jmedchem.5c00645

**Published:** 2025-04-07

**Authors:** Karolina Trepczyk, Safak Er, Irena Hlushchuk, Mikko Airavaara, Anna Alwani, Katarzyna Maziarz, Piotr Chmielarz, Kinga Słomska, Ewa Wieczerzak, Elżbieta Jankowska

**Affiliations:** † Department of Biomedical Chemistry, Faculty of Chemistry, University of Gdansk, Wita Stwosza 63, 80-308 Gdańsk, Poland; ‡ Pharmacology and Drug Development Division of Pharmacology and Pharmacotherapy, Faculty of Pharmacy, University of Helsinki, FI-00014 Helsinki, Finland; § Department of Brain Biochemistry, Maj Institute of Pharmacology, Polish Academy of Sciences, Smętna 12, 31-343 Kraków, Poland

## Abstract

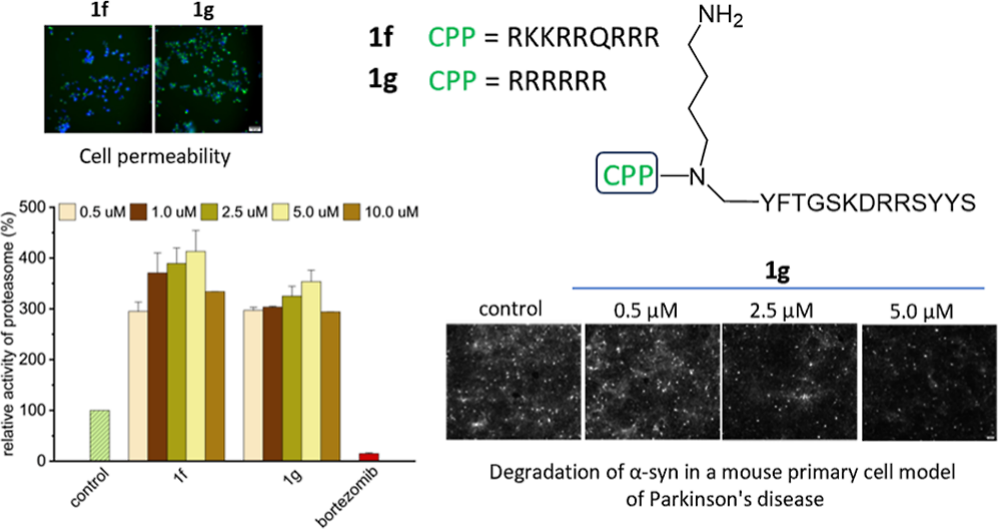

The development of age-related neurodegenerative diseases is associated with the accumulation of damaged and misfolded proteins. Such proteins are eliminated from cells by proteolytic systems, mainly by 20S proteasomes, whose activity declines with age. Its stimulation has been recognized as a promising approach to delay the onset or ameliorate the symptoms of neurodegenerative disorders. Here we present peptidomimetics that are very effective in stimulating the proteasome in biochemical assays and in cell culture. They are stable in human plasma and capable of penetrating the cell membranes. The activators demonstrated the ability to enhance h20S degradation of α-synuclein and tau, whose aggregates are involved in the development of Parkinson’s and Alzheimer’s diseases, respectively. The peptidomimetics did not show cytotoxicity to HEK293T and primary hippocampal cells. Additionally, these compounds were highly effective in reducing the amount of phosphorylated α-synuclein aggregates in hippocampal neurons in a mouse embryonic cell model.

## Introduction

Protein conformational diseases, such as Alzheimer’s disease (AD) and Parkinson’s disease (PD), due to difficulties in their early detection, the lack of effective forms of treatment, and the costs of care, constitute a serious global socio-economic problem. The occurrence of these diseases is strongly related to age and characterized by progressive neurodegeneration and dementia.^1^ Their common characteristic feature is the deposition of damaged proteins.^2,3^ In healthy cells, such proteins are eliminated by proteolytic systems, mainly by the 20S proteasome, which is responsible for the cellular housekeeping chores, degrading mutated, misfolded, and oxidized proteins.^4,5^ However, with age, the activity of this multienzyme weakens, resulting in impaired proteolysis and accumulation of damaged proteins.^6^

The eukaryotic 20S proteasome is a cylindrical complex composed of four stacked heptameric rings, with each ring containing seven distinct subunits of either the α or β type, arranged in an αββα configuration.^7,8^ The internal chamber created by these rings houses six active sites that mediate three distinct proteolytic activities: caspase-like (C-L), linked to the β1/β1′ subunits; trypsin-like (T-L), associated with the β2/β2′ subunits; and chymotrypsin-like (ChT-L), performed by the β5/β5′ subunits. In its inactive, or latent, state, the 20S proteasome restricts substrate access to the catalytic core through a gate formed by the N-terminal regions of the α subunits.^9^ Attachment of activating proteins, the dome-shaped heptameric 11S (PA28) activator complex,^10^ the homomeric activator PA200,^11^ or the ATP-dependent multimeric 19S regulatory cap,^12^ is needed to open the gate.

The results of recent studies argue that stimulating the proteasome can prevent the accumulation of damaged proteins and may be an effective therapeutic strategy.^13^ Over the past few years, several small molecules have been identified as proteasome activity enhancers. Among these, chlorpromazine^14^ and the imidazoline derivative TCH-165^15^ were shown in vitro to boost the activity of the 20S proteasome, facilitating the degradation of α-synuclein and tau proteins, which are associated with Parkinson’s and Alzheimer’s diseases, respectively. Additionally, two other compounds, AM-404 and MK-886, were found to promote the breakdown of α-synuclein in HEK293T cells.^16^ Furthermore, Liao et al. demonstrated that MK-886 enhanced proteasome function in living cells by monitoring the cleavage of GFP from a tau-GFP fusion protein expressed in HEK293T cells.^17^ Also, synthetic fluspirylene analogues were able to increase the proteolytic activity of the 20S proteasome. Fluspirylene and its derivative acylfluspirylene activated all three catalytic sites and prevented the aggregation and oligomerization of intrinsically disordered proteins.^18^

Peptides and peptidomimetics, whose advantages as therapeutic agents include primarily high affinity and specificity and low toxicity, constitute a separate group of proteasome activators. To this group belongs a synthetic PAP1 peptide, which increases chymotrypsin-like activity through a proteasome gate opening mechanism.^19^ This peptide protected fibroblasts from oxidative stress induced by hydrogen peroxide. It also prevented superoxide dismutase 1 (SOD1) aggregation in a cellular model of amyotrophic lateral sclerosis (ALS). Other compounds that increase the proteolytic activity of the proteasome are peptidomimetics TAT1–8,9TOD, and TAT1-DEN, which we developed based on the HIV-1 transcription activator TAT and its proteasome-binding RTP motif, also common to the 11S activators.^20^ TAT1–8,9TOD, and TAT1-DEN strongly stimulated ChT-L activity of h20S and activated the proteasome 8- and 10-fold, respectively. EC_50_ values for these compounds were in the range of 200–400 nM. The advantage of these compounds is their long half-life in human plasma and the ability to penetrate the blood–brain barrier. Moreover, these activators alleviated Alzheimer’s disease-like pathologies in model organisms, producing effects comparable to those generated by genetic proteasome augmentation.^21^ Another interesting group of activators includes compounds that we designed on the basis of the C-terminal fragment of the Blm10 protein, which is the yeast counterpart of the human PA200 activator. These compounds share with Blm10 the HbYX motif (Hb-hydrophobic, Y-tyrosine, X-any amino acid at the very C-terminus), which was demonstrated as responsible for anchoring them in the pocket between the α5 and α6 subunits of the proteasome.^22,23^ As we recently reported, the introduction of the HbYX motif into the sequence of proline- and arginine-rich (PR) peptides, known as proteasome inhibitors, converted these compounds into potent 20S activators that also bind in the α5-α6 pocket.^24^ However, while indispensable for activation, the HbYX motif alone is not sufficient to fully control the proteasome activity. We demonstrated that modification of the upstream regions of Blm analogues had a significant impact on the ability of these compounds to stimulate both the proteasome activity in HEK293-T cell lysates and the degradation of α-synuclein or the oxidized form of enolase.^25^ To better harness the potential of both the HbYX motif and the extended N-terminal region, we employed molecular modeling and designed peptides capable of binding not only within the canonical α5-α6 pocket but also in additional sites. This multivalent binding was expected to enhance interactions with the α subunits of the human 20S proteasome, leading to a more effective enzyme activation. Our strategy proved successful: X-ray crystallography revealed that one of the most potent Blm-based activators developed to date bound at three distinct pockets between α subunits.^26^ However, despite their promising ability to stimulate the proteasome, the peptide Blm activators have two major drawbacks: a lack of proteolytic stability and poor membrane permeability. In this work, we show the results of our efforts to obtain proteasome activators devoid of these drawbacks. These new compounds were stable under proteolytic conditions and effectively crossed the blood–brain barrier, simultaneously having an enhanced ability to stimulate the proteasome activity in the cell lysate. Moreover, the compounds stimulated the proteasome to degrade model protein substrates involved in the development of neurodegenerative diseases, α-synuclein, and tau protein. In studies conducted with a mouse embryonic cell model, the activators effectively reduced the level of α-synuclein aggregates in the hippocampal neurons.

## Results and Discussion

### Design and Synthesis of the 20S Modulators

In designing the new modulators, we based our approach on the sequences of two peptides, KYFTGSKDWRSYYS (compound **1**) and KYFTGSKDYRRYYS (compound **2**), which have demonstrated high efficacy in stimulating h20S activity.^26^ A common strategy to enhance peptide resistance to proteolysis involves replacing natural amino acids with their non-natural analogues. However, in our studies concerning peptide activators of the h20S proteasome, we found that substitutions with unnatural amino acids, such as 4-fluorophenylalanine or nitroarginine, do not significantly improve stability (Table S1). Therefore, aiming to obtain more stable analogues of **1** and **2**, we decided to modify the peptide skeleton. The insertion positions for these modifications were chosen based on the modulators’ digestion sites, identified by mass spectrometry. Two types of modifications were chosen: methylation of the backbone nitrogen and transfer to this atom of an amino acid side chain, which allows constructing a peptoid bond (Table 1). Either a native side chain (4-aminobutyl corresponding to the side chain of Lys) or the relevant isosteres (2-hydroxyethyl and 4-methoxybenzyl being the isosteres of the side chains of Ser and Tyr, respectively) were employed to obtain peptoids. We designed activators **1a**, **1e**, **2a**, **2b**, **2c**, and **2d** to verify how the ability to activate the proteasome will be affected by modifications present in the N-terminal region. To check how the activity of the 20S proteasome will be affected by compounds with modifications introduced in the C-terminal fragment, we designed modulators **1b**, **1d**, and **2e**. We also designed analogues possessing both modifications simultaneously (**1c**, **2f**). In this way, we obtained 11 new Blm activators. All compounds had the HbYX motif preserved.

**Table 1:**
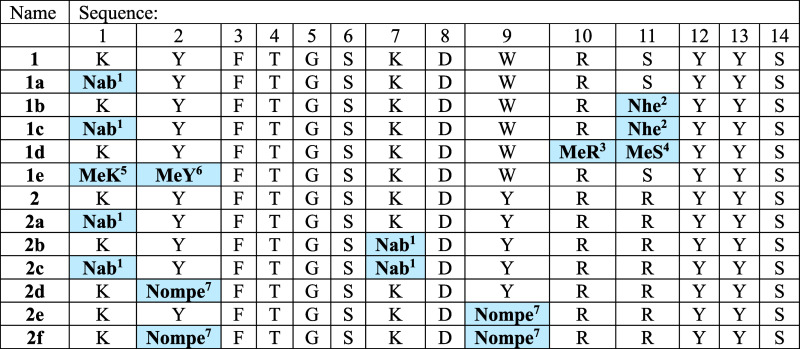
Names and Sequences of the Obtained Blm Peptides and Peptidomimetics^a^^,^^b^

a ^1^*N*-(4-aminobutyl)glycine. ^2^*N*-(2-hydroxyethyl)glycine. ^3^*N*-methylarginine. ^4^*N*-methylserine. ^5^*N*-methyllysine. ^6^*N*-methyltyrosine. ^7^*N*-(4-methoxybenzyl)glycine.

b The site of modification is marked by bold letters on the blue background.

**Scheme 1:**
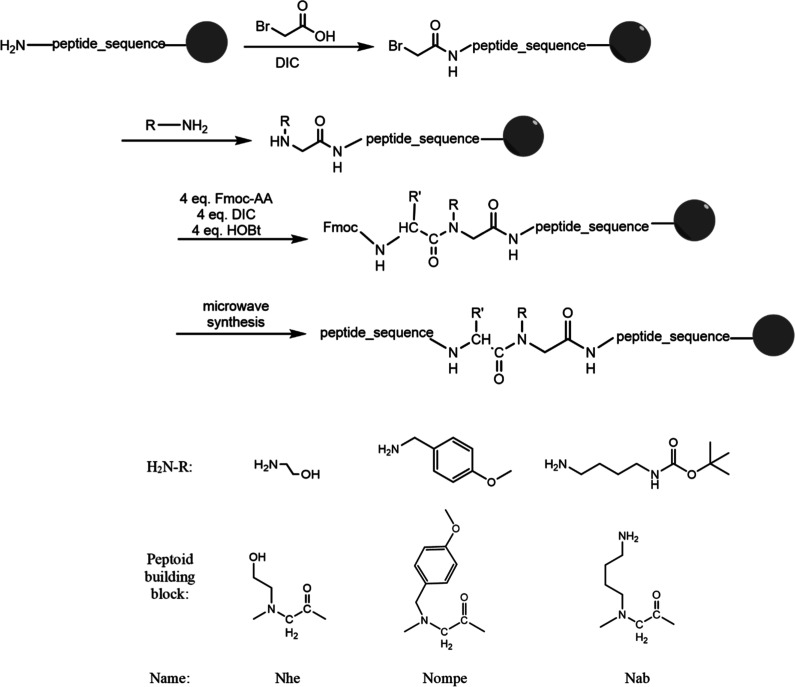
Scheme of the Synthesis of Activators Containing a Peptoid Bond

A peptoid bond incorporation was accomplished by utilizing bromoacetic acid and an appropriate amine. In contrast to the assembly of peptide fragments, the coupling reactions of the reagents forming the peptoid bond were carried out without the use of microwaves (Scheme 1). In the case of Fmoc-derivatives of N-methylated amino acids, they were introduced using a highly efficient coupling reagent (COMU) and extended coupling time (3 h), at room temperature, also without microwave application.

### Stability of the Compounds

**Figure 1:**
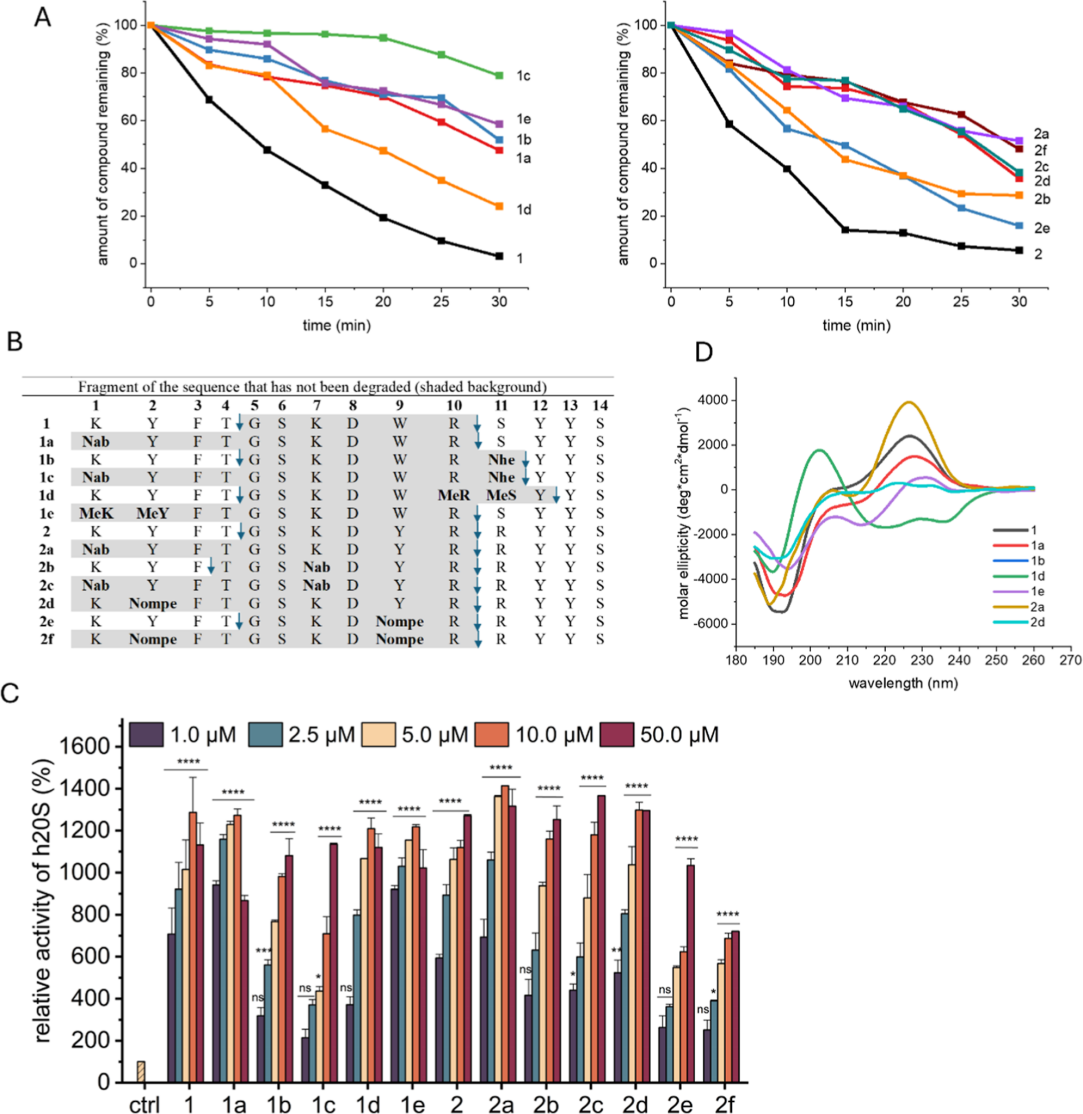
(A) Progress of the activator degradation during incubation of Blm peptidomimetics with human plasma, (B) degradation sites in the activators, identified by LC–MS. The sites of proteolytic digestion are marked by arrows. (C) Influence of the activators on the proteasome activity, probed with 15 μM Dabcyl-EDANS substrate. The relative activity was calculated by comparing the fluorescence increments in the presence of modulators with the values obtained for the latent proteasome (control), which were taken as 100%. The results are presented as the means ± SEM, number of repetitions *n* = 4. Statistics were performed using the one-way ANOVA test: *p < 0.05, **p < 0.01, ***p < 0.001, ****p < 0.0001, ns—no statistical significance. (D) CD spectra of the activators dissolved in water at a concentration of 0.2 mg/mL.

We tested the proteolytic stability of the obtained peptidomimetics by incubating them with human plasma at 37 °C for 30 min. The process was monitored by UHPLC. Using mass spectrometry, the proteolytic degradation sites were determined (Figure 1B). After 30 min of incubation, the amount of most peptidomimetics remained at the level of approximately 50–60%, while for their parent peptides **1** and **2**, it did not exceed 10% (Figure 1A). Apparently, the modifications made the modulators considerably less susceptible to the action of proteases present in the plasma. The most stable compounds **1c** and **2f** had modifications located in both the N- and C-terminal regions. Surprisingly, **2a** with a single modification at the N-terminus was also a very stable compound. The lowest proteolytic stability was demonstrated by **1d** with two N-methylated amino acids at the C-terminus and **2e** with a peptoid bond at position 9 (<30% of the modulator remained after 30 min of incubation). It should be noted that the introduced modifications affected the degradation sites not only in the immediate vicinity of the modification but also blocked the degradation of the peptide bonds further in the sequence.

### Activating Capacity of the Obtained Peptidomimetics

To assess the influence of substitutions increasing proteolytic stability on the modulators’ capacity to stimulate chymotrypsin-like, trypsin-like, and caspase-like activity of h20S, we used classic fluorogenic substrates: Suc-LLVY-AMC, Boc-LRR-AMC, and Z-LLE-AMC, respectively. To confirm the observed stimulating capacity, we also conducted assays using a FRET-type reporter peptide with a sequence consisting of 11 residues (Lys(Dabcyl)-Met-Ser-Gly-Phe-Ala-Ala-Thr-Ala-Glu(EDANS)-Gly).^27^ As we checked, the activated 20S hydrolyzed this substrate after more than one residue (Lys, Gly, and Glu), which makes it a probe better reflecting the concerted action of the proteasome’s three peptidases. Of the analogues of compound **1**, **1a** and **1e** showed similar enzyme-stimulating activity, reaching 12-fold when probed using Dabcyl-EDANS (Figure 1C) and being 8-fold for Suc-LLVY-AMC (Figure S1). The discrepancy in the level of activation was mainly due to the greater sensitivity of the FRET substrate, as it is not digested, unlike the AMC-based probes, by the latent proteasome, which results in greater differences in fluorescence relative to the controls. Compounds **1b**, **1c**, and **1d** exhibited lower capacity of activating h20S, which was evident across the entire concentration range when probed with Suc-LLVY-AMC (Figure S1). In contrast, in tests using Dabcyl-EDANS, **1d** at higher concentrations reached stimulation levels equal to those of the best activators, **1a** and **1e** (Figure 1C). Concomitantly, **1d** was a poor activator of T-L (Figure S2) and only a moderate activator of C-L (Figure S3). All these facts together suggest that **1d** may also stimulate noncanonical proteasome activity, namely, hydrolysis of bonds after small, neutral amino acids (SNAP), such as the Gly–Phe bond in Dabcyl-EDANS, which was confirmed as a digestion site by mass spectrometry. In the second series of compounds, parent peptide **2** and peptidomimetic **2a** were consistently indicated by both Suc-LLVY-AMC and Dabcyl-EDANS probes as the most effective h20S stimulators, especially when their lower concentrations were considered. Similar consistency was observed for **2e** and **2f**, which were indicated by both substrates as the least effective stimulants. These compounds were also the weakest activators of the T-L and C-L peptidases. Modulators **2b**, **2c**, and **2d** showed comparable stimulatory capacity when tested with Dabcyl-EDANS, Boc-LRR-AMC, and Z-LLE-AMC, while they clearly differed in activity when probed with Suc-LLVY-AMC. At a 1 μM concentration, **2c** and **2d** activated ChT-L peptidase with the same moderate efficiency, whereas at higher concentrations, **2b** and **2c** stimulated it with efficiency comparable to **2** and **2a**, while **2d** was as inefficient as **2e**. The ability to affect individual peptidases of the proteasome in different ways is most likely related to the allosteric mechanism of action of the modulators. Binding at a site distant from the catalytic compartment may result in greater diversity in the pathways by which the signal reaches active sites and differential effects of that signal on each peptidase.

Although all modulators share an identical HbYX motif, they stimulate proteasome peptidases differently, highlighting the role of the sequence upstream of the HbYX in tuning the enzyme activity. As can be seen in Figure 1C, introducing modifications in the region immediately adjacent to the HbYX did not produce good results. Compounds **1b**, **1c**, and **1d** stimulated the proteasome less strongly than **1**, especially at low concentrations. Modifications introduced at the N-terminus yielded better results as both **1a** and **1e** did not lose their ability to effectively stimulate h20S. Moreover, these compounds at the lowest concentrations were even more potent than **1** (Figure 1C). In the case of peptidomimetics from the second series, **2e** and **2f**, having 4-methoxybenzylglycine instead of the Tyr residue at position 2 and/or 9, were the least effective in stimulating the proteasome to digest the Dabcyl-EDANS substrate. Although none of these substitutions directly affected the HbYX motif or were in close proximity to it, they significantly impaired the stimulatory capacity. In contrast, compound **2a**, in which the Lys1 residue was replaced by a peptoid moiety, proved to be an effective activator.

Using circular dichroism experiments, it was not possible to directly relate the activation properties of the modulators to their secondary structure. Most peptidomimetics were characterized by a disordered structure indicated by a clear minimum in the range of 190–200 nm and a maximum in the vicinity of 225–235 nm (Figure 1D). The only exception was compound **1d** with N-methylated amino acids present in the C-terminal sequence. The spectrum of this compound has a maximum in the range of 200–210 nm and two minima at about 220 and 235 nm. The shape of the spectrum corresponds to an α-helix, but the bathochromic shift may indicate the presence of β-bends.

### Development of Cell-Permeable Modulators

**Figure 2:**
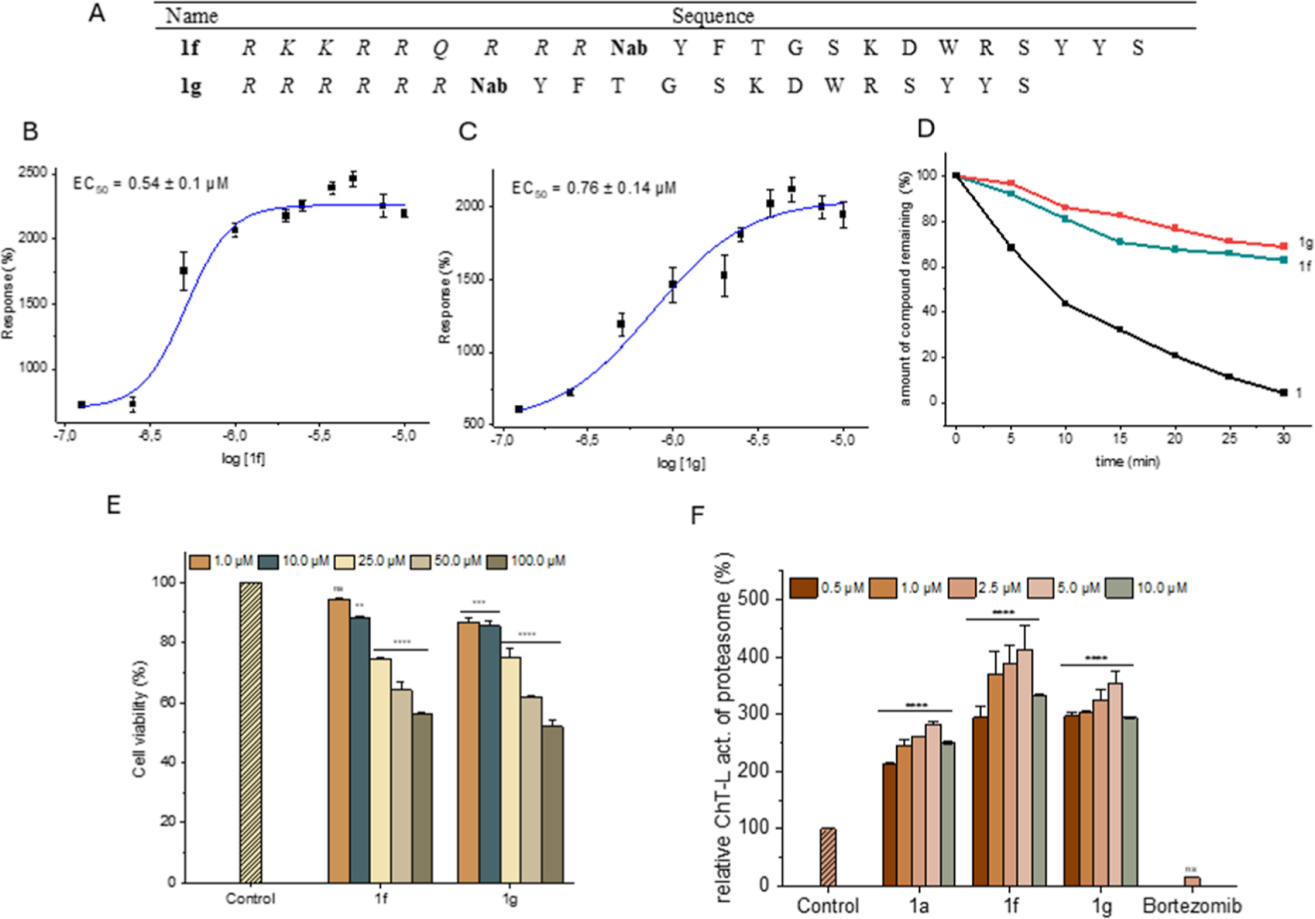
(A) Names and sequences of the activators with the CPP sequence attached (Nab—*N*-(4-aminobutyl)glycine). (B) Stimulating effect of **1f** on the ChT-L activity of h20S proteasome, probed with the Suc-LLVY-AMC substrate, *n* = 6. (C) Stimulating effect of **1g** on the ChT-L activity of h20S, probed with Suc-LLVY-AMC, *n* = 6. (D) Stability in human plasma of **1f** and **1g** in comparison to their parent compound **1**. (E) Cytotoxicity of **1f** and **1g** against the HEK293T cell line, *n* = 3. The cells were incubated with the activators for 24 h, and then the MTT test was performed. (F) Stimulating effect of **1a**, **1f**, and **1g** on proteasome activity in the HEK293T cell lysate, probed with the Suc-LLVY-AMC substrate, *n* = 4. The results are presented as the means ± SEM. Statistical analysis was performed by comparing the obtained results with the control using the one-way ANOVA test: *p < 0.05, **p < 0.01, ***p < 0.001, ****p < 0.0001, ns—no statistical significance.

Considering simultaneously the stability of the compounds in plasma (Figure 1A) and their ability to stimulate degradation of classic fluorogenic substrates (Figures S1–S3) and FRET-type Dabcyl-EDANS probe (Figure 1C), we selected peptidomimetic **1a** to introduce modifications improving the compound’s cell permeability. The ability to penetrate the cell membrane is essential for the compound to be used as a therapeutic agent, and this can be ensured by attaching a cell-penetrating peptide (CPP) to the pharmacophore. The database of CPP sequences of various types is available in the literature. For instance, it has been described that a peptide consisting of more than five residues with a guanidinium moiety effectively crosses the cell membrane.^28^ Also, the 48–58 fragment of the HIV-1 Tat protein was found to have CPP properties. We decided to use a fragment of this peptide, with the sequence RKKRRQRRR (tat), and a peptide consisting of six arginine residues (6r) as promoters of cell membrane penetration (Figure 2A).

The capacity of the two peptidomimetic-CPP constructs to activate the h20S proteasome was tested using standard fluorogenic substrates. These studies revealed that stimulating the ability benefited from the extension of the modulator sequence by CPP (Figure 2B,C). The EC_50_ (the concentration causing 50% growth of activity compared to the vehicle-treated control) was below 1 μM for both activators. Moreover, **1f**, already at 1 μM concentration, stimulated the ChT-L peptidase approximately 16-fold (Figure S4). Containing the 6r sequence, **1g** was slightly less effective in activating this peptidase but with a greater efficiency affected the T-L. A control study of the CPPs alone showed that the stimulating effect disappeared without the presence of the **1a** modulator (Figure S4). Moreover, increasing the concentration of the tat peptide even resulted in proteasome inhibition. Interestingly, in the case of T-L peptidase, a reversal of stimulation was observed. Unlike their parent compound **1a**, **1f** and **1g** constructs activated the T-L less and less with an increasing concentration. This was probably due to the accumulation of basic residues at the N-terminus, which led to the modulators effectively competing with the substrate of the T-L peptidase. Although bulkiness most likely does not allow the constructs to freely enter the latent proteasome, activation of the enzyme owing to the modulator binding could change this situation. The binding-induced opening of the gate probably facilitated the entry to the catalytic chamber not only of the Boc-LRR-AMC substrate but also of unbound molecules of **1f** and **1g**. As a result, their degradation interfered with the fluorescent substrate proteolysis. We confirmed this hypothesis by MS analysis carried out after the incubation of **1f** and **1g** with h20S, observing signals corresponding to the molecular weight of the compounds with arginine residues excised from the sequence. Nevertheless, the addition of the CPP sequence did not impair the stability of the modulators in plasma. After 30 min of incubation, still more than 60% of the compound was intact (Figure 2D).

We also tested the effect of the modulator-CPP constructs on the cell culture viability. For this purpose, we performed the MTT assay (Figure 2E). Using this assay, we examined the metabolic activity of cells by measuring the absorbance of the MTT metabolite at a wavelength of 570 nm. At the highest tested concentration of the activators, cell viability was maintained at approximately 50%, which indicated that the compounds exhibit moderate cytotoxicity. At the lower concentrations of 1 and 10 μM, the modulators were not cytotoxic; therefore, this was the concentration range that was used in subsequent cellular studies.

**Figure 3:**
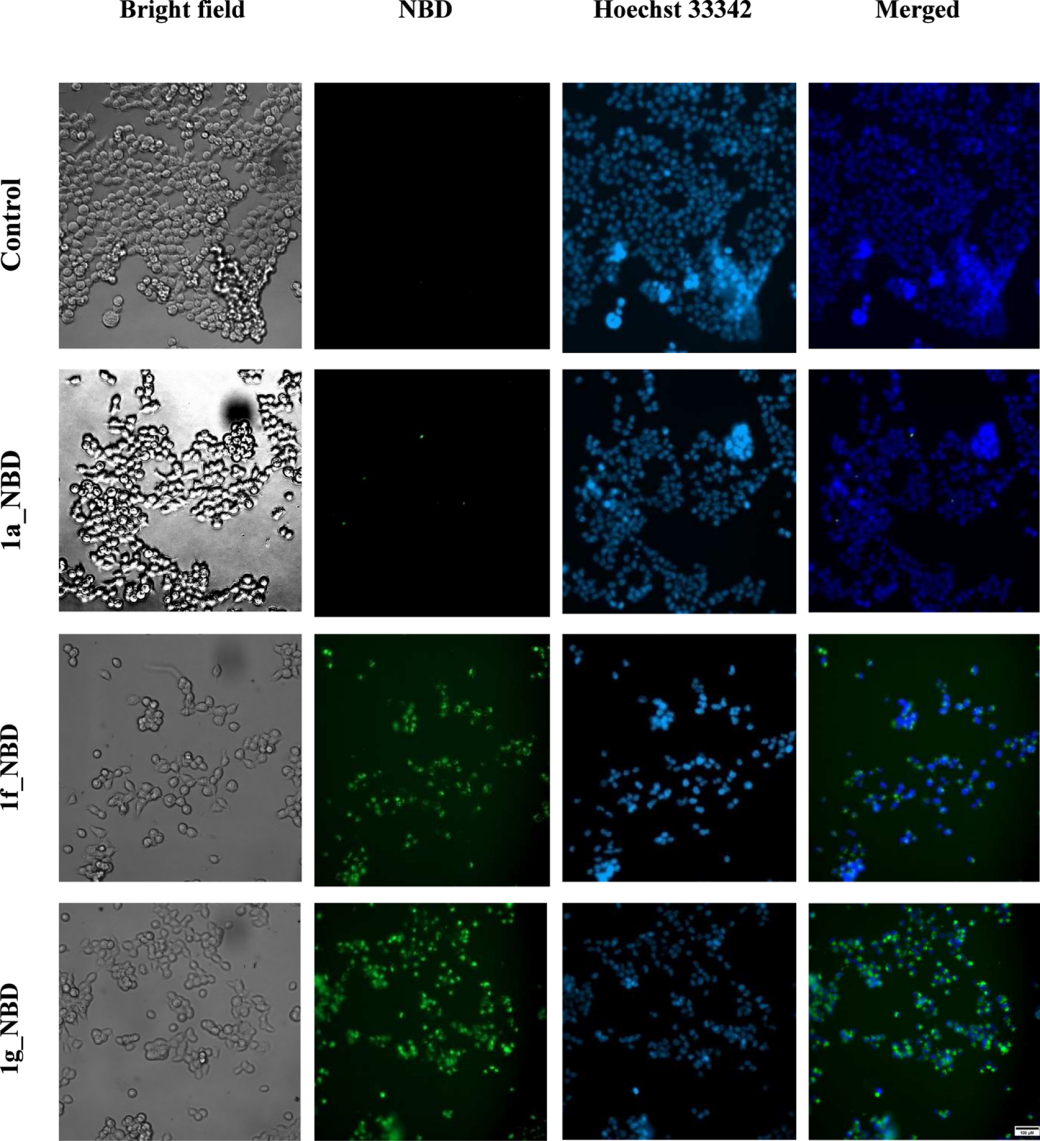
Microscopic images of HEK293T cells incubated for 3 h with the activators labeled with the NBD fluorescence tag. Columns from left to right present: bright-field images showing cell morphology; fluorescence of NBD-tagged compounds, indicating the penetration of these compounds into cells (green fluorescence); fluorescence of Hoechst 33342 dye, showing its binding to DNA and allowing visualization of cell nuclei (blue fluorescence); superimposition of NBD (green) and Hoechst 33342 (blue) fluorescence images, showing the localization of NBD compounds in relation to cell nuclei. The figure shows representative images selected from three independent replicates. The scale is 100 μm.

In order to verify the permeability of the constructs across cell membranes, we synthesized their analogues with an NBD fluorophore attached at the N-terminus. The excitation wavelength for this fluorophore is 467 nm, and the emission wavelength is 539 nm. Figure 3 shows fluorescence microscopy images of cells treated with the labeled activators. In the case of the control and compound **1a_NBD**, no fluorescence is noticeable in the images taken by using the Alexa Fluor 488 filter (column marked as NBD). However, for the modulators with the CPP sequence, fluorescence from the attached NBD tag is visible inside HEK293T cells, demonstrating that the incorporation of the CPP sequence improved the permeability of the modulators across the cell membrane. Comparing the images for compounds **1f_NBD** and **1g_NBD**, we noticed a small difference in the intensity of the observed green fluorescence, which indicates that the ability of **1g** to penetrate the cell membrane may be slightly higher than that of **1f**. The experiment with Hoechst 33342 confirms the results obtained in cytotoxicity tests since staining with this reagent proved the presence of living cells after their treatment with compounds **1f** and **1g**.

To assess whether activators are able to stimulate the proteasome in the cellular environment, we conducted experiments using the HEK293T cell lysate. Modulators with the CPP sequence attached at a concentration of 5 μM activated the proteasome approximately 4-fold. These studies confirmed that **1f** and **1g** had a higher efficacy than their parent compound **1a** (Figure 2F).

### Binding of the Modulators with h20S

**Figure 4:**
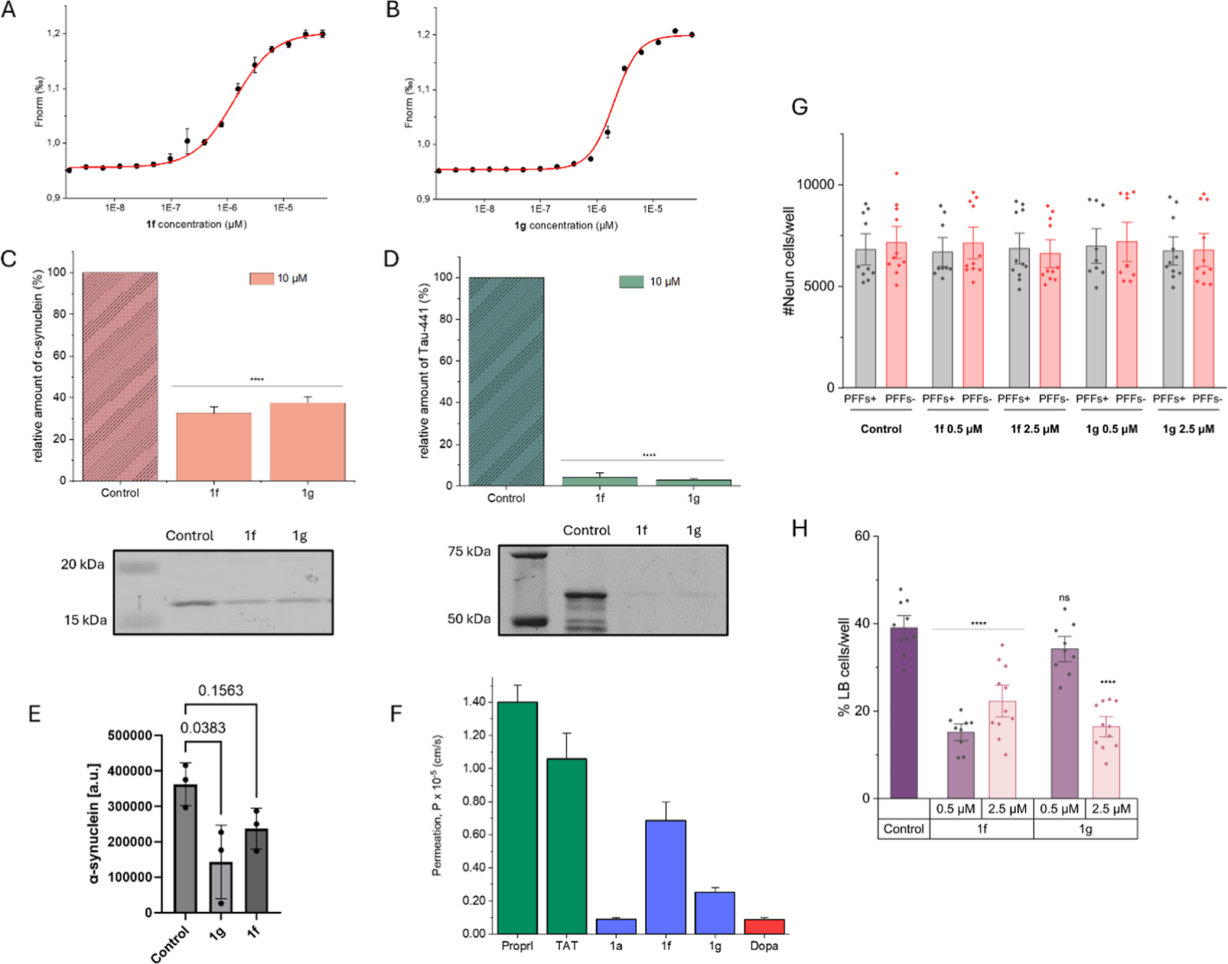
(A,B) Binding of activators (A: **1f**, B: **1g**) to the fluorescently labeled proteasome, determined by the MST technique. Number of biological replicates *n* = 3. Effect of activators on the degradation of (C) α-synuclein and (D) Tau-441 protein. The graphs show the relative amount of a protein substrate remaining after incubation with the h20S proteasome, without the presence of activators (control) and in their presence at a concentration of 10 μM. Number of biological replicates *n* = 3. Example electropherograms are presented below the graphs. (E) Amount of α-synuclein remaining after 7 days of treating 14 day-old primary cortical cultures with 2.5 μM activators; *n* = 3 biological replicates. (F) Permeability of **1f** and **1g** across the blood–brain barrier determined using the PAMPA test. Number of biological replicates *n* = 3. Tat47–57 (TAT) and propranolol (Proprl) were positive controls, while dopamine (Dopa) was used as a negative control. (G) Survival of matured hippocampal cells after 7 days of incubation with the activators, demonstrating the absence of cytotoxic effects. PFFs– and PFFs + denote control cells and cells to which α-synuclein preformed fibrils were added, respectively. (H) Number of hippocampal neurons containing Lewy body-like aggregates after 7 days of incubation with the activator or without the addition of modulators (control). The number of biological replicates in G and H was 3, and the number of technical replicates was 9–11. The results in C, D, and F are presented as the means ± SEM. The results in E, G, and H are presented as the means ± SD. Statistical analysis was performed by comparing the obtained results with the control using the one-way ANOVA test: *p < 0.05, **p < 0.01, ***p < 0.001, ****p < 0.0001, ns—no statistical significance. Ability of the modulators to cross the blood–brain barrier.

To further characterize **1f** and **1g** activators, we conducted a series of assays. One of them tested the ability of the compounds to interact with the human 20S proteasome. We used microscale thermophoresis for this purpose and performed measurements utilizing proteasomes labeled with the NT-647 dye. Using the recorded thermophoretic transitions, we determined proteasome-modulator affinity (Figure 4A,B). The results confirm that the activators interact with the 20S proteasome. The EC_50_ for **1f** is 1.33 ± 0.09 μM, while for **1g**, it is 2.03 ± 0.14 μM.

### Influence of the Modulators on the Degradation of Native Proteins

The effectiveness of the compounds has also been examined by using proteins as substrates. By means of SDS-PAGE electrophoresis, we checked the extent to which model proteins were digested by the 20S proteasome alone and after its stimulation by 10 μM modulators. Under the experimental conditions, compounds **1f** and **1g** significantly improved the efficiency of α-synuclein degradation by the proteasome (Figures 4C and S5). After incubation with the enzyme in the presence of each modulator, less than 40% of synuclein remained. An even higher efficiency was observed in the case of Tau protein, which was digested in more than 90% yield in the presence of the modulators (Figures 4D and S5). The activators were also able to reduce the amount of endogenous α-synuclein in primary cortical cultures (Figure 4E), although the effect reached statistical significance only for **1g**.

In neurodegenerative diseases, proteins prone to aggregation accumulate in the neuronal cells. Therefore, a necessary feature of potential drugs is not only permeability across the cell membrane but also the ability to cross the blood–brain barrier (BBB). To preliminarily investigate this feature, we performed parallel artificial membrane permeation assays (PAMPA) including as positive controls propranolol and Tat47–57, known for their ability to penetrate the blood–brain barrier. Dopamine hydrobromide was included as a negative control. For comparison, we also tested compound **1a**, which is devoid of a CPP sequence. The results demonstrated that both **1f** and **1g** had the ability to penetrate the artificial membrane mimicking the BBB, with **1f** being more than 2.5 times better than **1g** (Figure 4F). In contrast, the permeability of compound **1a** was at the level of a negative control, which confirms that CPP can significantly improve the druggability of proteasome modulators.

### Ability of the Modulators to Enhance Degradation of α-Synuclein in a Mouse Cell Model of Parkinson’s Disease

**Figure 5:**
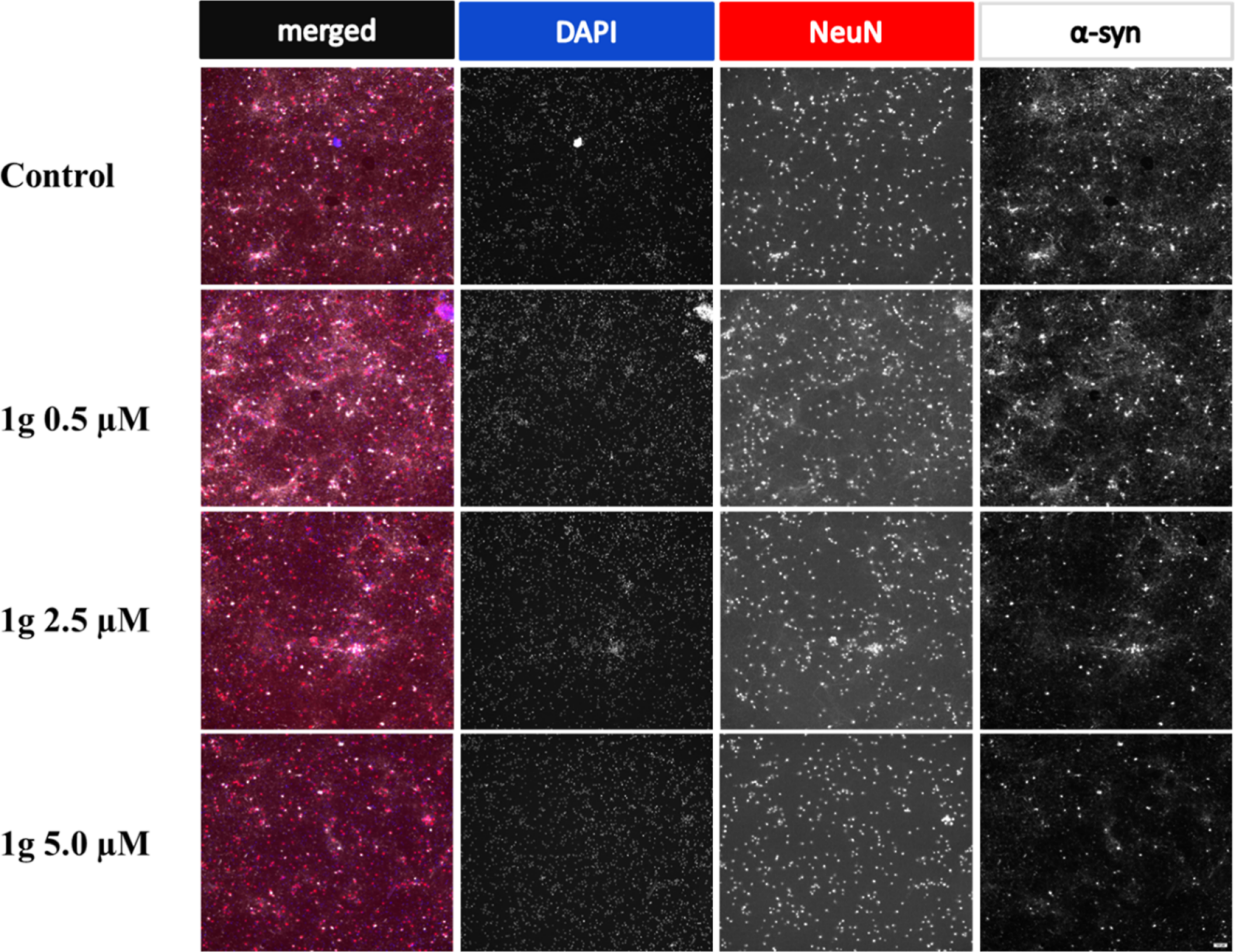
Comparison of microscopic images of hippocampal cells incubated without an activator and in the presence of **1g** at three different concentrations. The figure shows representative images selected from three independent replicates. The scale is 100 μm. Images were obtained from immunostaining with DAPI (nuclei, blue), NeuN (neuronal marker, red), and antibody detecting phosphorylated α-synuclein (pS129-αsyn, white). The overlay of signals from all detections (first column) indicates the localization of pS129-αsyn in neurons, while immunofluorescence of α-syn (last column) demonstrates that the amount of the aggregation-prone protein decreases as the concentration of **1g** increases.

Encouraged by the results received, we tested the ability of the modulator-CPP constructs to increase α-synuclein degradation in a cell model using primary mouse neuronal cells. The presence of Lewy-like neurites and Lewy-like bodies containing aggregated α-synuclein with phosphorylated serine 129 (pS129-αsyn) is a characteristic feature of the brains of people with Parkinson’s disease. To mimic these aggregations, we introduced a solution of preformed fibrils (PFFs) to the media of neuronal cells, which led to the induction of α-synuclein phosphorylation and its aggregation in neurons isolated from mouse embryos. PFFs are purified, prion-like fibrils of α-synuclein that are produced by expressing a recombinant monomeric protein in bacteria. Seven days after PFFs are introduced to the cells, large aggregates are visible in the cell soma, often next to the cell nucleus. We added the PFF solution to the hippocampal cells, and after an hour of incubation, we introduced solutions of the activator. After 7 days, we immunostained neuronal cells using a monoclonal antibody against the NeuN protein, which is commonly used in neuronal differentiation studies to assess their functional status. Additionally, we stained the nuclei of all incubated cells with DAPI. As can be seen in Figure 4G, the tested concentrations of the activators did not show cytotoxicity to hippocampal cells. It is noteworthy that the number of cells in samples with activators was slightly higher (Figure 5), which may indicate neuroprotective properties. This ability may prove crucial in eliminating nonmotor symptoms of Parkinson’s disease. The tested activators effectively reduced the number of hippocampal cells containing α-synuclein (Figure 4H) without decreasing the total number of neurons (Figure 4G). The main tool for monitoring this process was an antibody against pS129-αsyn. For **1g**, there is a visible dependence of the degradation ability on concentration, while **1f** even at a very low concentration reduced by 50% the number of aggregates formed. The fluorescent microscopy images (Figure 5) show the difference between the amounts of aggregated protein in the control sample and in the samples containing activators at different concentrations.

The ability to reduce α-synuclein aggregation in fibril-treated neurons corroborates the observed capacity of proteasome activators to increase α-synuclein degradation in vitro (Figure 4C) and reduce endogenous α-synuclein levels in cultured cortical neurons (Figure 4E). It has been proposed that the proteasome plays a role in degrading and maintaining proper levels of natively unfolded proteins, like α-synuclein,^29,30^ albeit it might also be involved in degradation of some misfolded α-synuclein species. Higher endogenous α-synuclein levels increase cells’ propensity to develop pathological aggregates,^31^ whereas reducing endogenous α-synuclein confers resistance to seeding by exogenously applied pathological α-synuclein fibrils.^32^ It has been proposed that cells with lower endogenous α-synuclein levels can effectively manage forming aggregates while those with high levels are quickly overwhelmed.^33^ Hence, age-related increases in α-synuclein levels are thought to predispose individuals to Parkinson’s disease (PD) and neurons with the highest α-synuclein levels are more affected by PD pathology.^34,35^ Our proteasome activators, by reducing α-synuclein levels, appear to protect neurons from fibril-induced aggregation, thus addressing a key mechanism in PD pathology. These findings, combined with the BBB permeability (Figure 4F) and lack of toxicity to neuronal cells (Figure 4G) of our compounds, support the potential of proteasome activators as a promising treatment strategy for PD.

## Conclusions

We described 11 peptidomimetic modulators with modifications improving their stability in plasma. The best of them displayed the ability to stimulate the ChT-L peptidase of the human 20S proteasome approximately 8–9 times, the T-L about 8 times, and the C-L about 24 times. In addition, the Dabcyl-EDANS probe, which better mimics protein substrates, was degraded in the presence of the most potent activators 12–14 times more efficiently. Attaching the CPP sequence to the selected activator resulted in compounds **1f** and **1g** that were able to penetrate the cell membrane, stimulated proteasome activity approximately 17 times, and were quite stable under proteolytic conditions. In addition, these activators were able to cross the artificial blood–brain barrier, as we proved in the PAMPA. The modulators demonstrated the ability to increase the efficiency of h20S degradation of model protein substrates, α-synuclein and tau, whose aggregates are involved in the development of neurodegenerative diseases. They were also able to effectively stimulate the proteasome in a cell culture and did not show cytotoxicity to both HEK293T cells and mouse embryonic hippocampal cells. Additionally, these compounds were highly effective in reducing the amount of phosphorylated α-synuclein in hippocampal cells in a mouse embryonic cell model. These results are promising and indicate that by stimulating the human proteasome with compounds such as **1f** and **1g**, it is possible to diminish the pathological effects of protein aggregates.

## Experimental Section

### General Methods

All reagents and solvents were obtained from commercial sources and used without further purification. Product purification was performed by semipreparative reverse-phase high-performance liquid chromatography (RP-HPLC), equipped with a Jupiter 4 μm Proteo column, 90 Å, 21.2 mm × 250 mm, 15 mL/min, 60 min gradient from 10% to 80% aqueous acetonitrile containing 0.1% TFA. The purity of final compounds was >95%, as determined by UHPLC analysis (Shimadzu, Tokyo, Japan) on a Kinetex column (2.1 mm × 100 mm, 2.6 μm, 100 Å (Phenomenex)), in a gradient from 5% to 80% aqueous acetonitrile solution containing 0.1% TFA, eluting at 0.5 mL/min. The identity of pure products was evaluated by ESI-IT-TOF LCMS (Prominence, Shimadzu) and/or MALDI-TOF MS (autoflex maX, Bruker, Billerica, MA, USA).

### Synthesis of Peptides

All peptides were synthesized on solid-phase supports (Wang, Cl-TCP(Cl) ProTide, or TentaGel R RAM resin) using standard Fmoc (9-fluorenylmethoxycarbonyl) chemistry. The synthesis was performed with a Liberty Blue microwave peptide synthesizer (CEM, Matthews, NC, USA). Fmoc-protected amino acids were coupled using a 1:1 solution of 0.5 M *N*,*N*′-diisopropylcarbodiimide (DIC) and 1 M ethyl cyano(hydroxyimino)acetate (Oxyma Pure) in dimethylformamide (DMF). Standard coupling cycles were as follows: 170 W, 75 °C, 15 s, and 30 W, 90 °C, 110 s. For compounds **1f** and **1g**, a 0.5 M solution of *O*-(1*H*-6-chlorobenzotriazole-1-yl)-1,1,3,3-tetramethyluronium hexafluorophosphate (HCTU) in DMF was used as a coupling reagent and 2 M *N*,*N*-diisopropylethylamine (DIPEA) in *N*-methylpyrrolidone (NMP) was utilized instead of Oxyma Pure. Modified microwave cycles were applied for these steps (29 W, 75 °C, 600 s).

### Synthesis of Peptidomimetics with Peptoid Bonds

The peptoid bond was introduced manually. A mixture (50:50, v/v) of 2 M bromoacetic acid and 2 M DIC activator in DMF was prepared. The mixture was shaken until a precipitate was formed and then added to the peptidyl resin. After 30 min, the solution was filtered off, the resin was washed with DMF, a 1.5 M solution of the appropriate amine in DMF was added, and then the mixture was shaken for another 90 min. The following amines were used to synthesize the peptidomimetics: *N*-Boc-1,4-diaminobutate (**1a**, **1c**, **2a**, **2b**, **2c**, **1f**, **1g**), 4-methoxybenzylamine (**2d**, **2e**, **2f**), and ethanolamine (**1b**, **1c**). Fmoc-amino acid immediately following the modification was also conjugated by manual synthesis, using 4 equiv of Fmoc-amino acid, DIC, and hydroxybenzotriazole (HOBt).

### Synthesis of Peptidomimetics with *N*-Methyl Amino Acid

Three equiv of Fmoc-*N*-methylated amino acid, 3 equiv of (1-cyano-2-ethoxy-2-oxoethylidenaminooxy)dimethylamino-morpholino-carbenium hexafluorophosphate (COMU), and 5.4 equiv of DIPEA dissolved in DMF were shaken for 3 min, and after this preactivation, the mixture was added to the peptidyl resin. The coupling reaction was carried out for 3 h at room temperature. A deprotection reaction was performed twice for 15 min, each time with 20% piperidine in DMF. The following standard N-protected amino acid was incorporated into the sequence according to the same protocol.

### NBD Attachment

The NBD fluorescent tag was attached to the N-terminal α-amino group of the lysine residue after the final deprotection and before the cleavage of the compounds from the solid support. The coupling was carried out by agitating 3 equiv of 4-chloro-7-nitrobenzofurazan and 3 equiv of DIPEA with the peptidyl resin in DMF for 24 h at room temperature.

### Enzymatic Activity Tests

#### Enzymatic Activity Test

The effect of all synthesized peptidomimetics on the catalytic activity of the proteasome was tested using a human proteasome isolated from erythrocytes. Suc-LLVY-AMC, Boc-LRR-AMC, and Z-LLE-AMC fluorogenic substrates (Bachem, Bubendorf, Switzerland) were used for assessment of the chymotrypsin-, trypsin-, and caspase-like activities, respectively. A homemade FRET-type substrate Dabcyl-EDANS (Lys(Dabcyl)-Met-Ser-Gly-Phe-Ala-Ala-Thr-Ala-Glu(EDANS)-Gly)^26^ was used as an additional probe. Stock solutions of the substrates and tested peptidomimetics were prepared in dimethyl sulfoxide (DMSO), ensuring that the final concentration of DMSO in all samples remained consistent at 2%. The small fluorogenic substrates were used at a final concentration of 100 μM, while Dabcyl-EDANS was prepared at a final concentration of 15 μM. Modulators were tested in concentrations ranging from 1.0 to 50 μM, with compounds containing TAT or 6R sequences tested at lower concentrations ranging from 0.5 to 10 μM. Activity tests were conducted in 96-well plates using 50 mM Tris/HCl, pH 8.0, as an assay buffer. The final concentration of h20S proteasome was adjusted to 0.002 mg/mL (2.8 nM). The fluorescence of released aminomethylcoumarin (AMC) was monitored at 460 nm (λex was 380 nm), while the hydrolysis of Dabcyl-EDANS was detected by measuring emission at 493 nm (λex was set at 335 nm). Fluorescence measurements were taken continuously every 2 min over a 60 min period at 37 °C using a Tecan Infinite M200Pro spectrofluorometer (Tecan Trading AG, Männedorf, Switzerland). All activity assays were performed in at least three independent replicates. The relative activity was calculated in relation to the catalytic activity of the vehicle (DMSO)-treated latent proteasome, which was regarded as 100%. Statistical analysis was performed using the one-way ANOVA followed by Tukey’s post-hoc test. *P*-value <0.05 was considered statistically significant.

#### Stability in Human Serum

The modulators were incubated with human serum (Merck, Darmstadt, Germany) at 37 °C for 30 min. The compounds were dissolved in water, and their final concentration was 200 μM. Every 5 min, samples were taken from the incubated solution, and the reaction was stopped with 6% trichloroacetic acid (TCA) added to the sample in a 1:1 (v/v) ratio. Next, the samples were centrifuged for 10 min at 4 °C and 14,000 rpm. The supernatant was subjected to HPLC and MS analyses. The progress of proteolytic degradation of modulators was monitored using a UHPLC chromatograph (Shimadzu, Tokyo, Japan) with a Kinetex analytical column 100 mm × 2.1 mm, 2.6 μm, 100 Å (Phenomenex, Torrance, CA, USA) with UV detection at a wavelength of 223 nm. The degree of degradation was estimated based on the area of the peaks corresponding to the starting materials. In order to identify peptide fragments resulting from degradation, analysis was performed using an Autoflex maX MALDI TOF mass spectrometer (Bruker, Torrance, CA, USA). All experiments were performed in at least three independent replicates.

#### Protein Substrate Degradation Assay

The experiment was performed as it was described.^36^ Briefly, α-synuclein (rPeptide, Watkinsville, GA, USA) and Tau-441 (Novus Biologicals, Centennial, CO, USA) were dissolved in 20 mM HEPES buffer (pH 7.4) and mixed with a human 20S proteasome. Samples were incubated at 37 °C for 1.5 h, after which the reaction was terminated by adding 4× Laemmli buffer. The levels of undigested proteins were evaluated by SDS-PAGE. The band intensities of proteins incubated with h20S in the presence of a modulator were compared to those of proteins incubated with the enzyme alone, which were set as 100%. Three independent experiments were performed for each protein. The results are presented as the means ± SD. One-way ANOVA with Tukey’s post-hoc test was used to determine statistical significance.

#### MTT Assay

The assay was performed as it was described.^36^ Briefly, human embryonic kidney cells (HEK293T) were cultured in complete medium for 2 days, after which the medium was replaced with the test compounds at concentrations of 1, 10, 25, 50, or 100 μM. The cells were incubated for 24 h and then treated with MTT (3-[4,5-dimethylthiazol-2-yl]-2,5-diphenyltetrazolium bromide; Acros Organics, Geel, Belgium). The formazan crystals were dissolved in DMSO, and the absorbance of the solution was measured at 570 nm. The means of three biological replicates are presented. Statistical analysis was performed by comparing the obtained results to the control using the one-way ANOVA test.

#### Proteasome Activity in the Cell Lysate

The experiment was carried out as it was described.^25^ Briefly, HEK293T cells were lysed, and the lysate was treated with the modulators at a concentration of 1, 2.5, 5, 10, and 25 μM, only in the case of modulators with the TAT or 6R sequence attached were the concentrations 0.5, 1, 2.5, 5, and 10 μM. The proteasome activity was probed using Suc-LLVY-AMC substrate. One μM bortezomib was used as a negative control. All experiments were performed in at least three independent replicates. One-way ANOVA with Tukey’s post-hoc test was used to determine statistical significance.

#### Fluorescence Microscopy

HEK293T cells were incubated in DMEM supplemented with 10% FBS and penicillin/streptomycin (100 units/mL/100 μg/mL), at 37 °C with 5% CO_2_. Cells were seeded on 24-well plates at a density of 3 × 10^4^ cells per well and incubated in 0.5 mL of the complete medium for 48 h. Then, the fresh medium containing the modulators labeled with NBD fluorophore was added to each well at a concentration of 10 μM. Half an hour before the end of the incubation, 20 μL of Hoechst 33342 dye was added to each well, and then the plate was placed back in the incubator. After the 3 h incubation, cells were washed thoroughly with PBS, and a phenol red-free culture medium (FluoroBriteTM DMEM, Gibco/Thermo Fisher Scientific, Waltham, MA, USA) was added. Subsequently, the cells were examined using a Zeiss AXIO Observer D1 microscope (Carl Zeiss AG, Oberkochen, Germany) using an Alexa Fluor 488 filter.

#### Parallel Artificial Membrane Permeability Assay

The PAMPA was performed as previously described.^21^ Briefly, a 2% solution of porcine brain lipid membrane (PBL; Avanti Polar Lipids Inc., Alabaster, AL, USA) in anhydrous dodecane was used as a mimic of the blood–brain barrier. Propranolol hydrochloride (Merck, Darmstadt, Germany) and Tat47–57 (synthesized in-house) served as positive controls, while dopamine hydrobromide (Merck, Darmstadt, Germany) was used as a negative control. Stock solutions of the tested compounds and controls were prepared at a concentration of 10 mM in deionized water and then diluted to 500 μM using 0.1 M PBS containing 5% DMSO. The donor–acceptor plate assembly was incubated at 37 °C while being shaken at 250 rpm for 22 h. Absorbance at 290 nm was measured for propranolol using an Infinite M200 Pro plate reader (Infinite M200 Pro plate reader, Tecan). For the other samples, fluorescence measurements were performed after the compounds were reacted with 0.02% fluorescamine. The rate of passive diffusion was calculated as the linear velocity of permeation (Pe). The data represent an average of three independent biological replicates.

#### Primary Neuronal Cultures Preparation

All animal experiments were performed following the protocols evaluated and approved by the Laboratory Animal Centre of the University of Helsinki (Ethics Approval License KEK24-013) and in accordance with the European Community Guidelines (Directive 2010/63/EU). The cortices were dissected from 16 to 17 day embryos (E16-E17) of Albino Swiss (CD1) mice, washed with HBSS, and incubated with trypsin for 17 min at 37 °C for dissociation. After the incubation, tissues were treated with DNase I in Hank’s Balanced Salt Solution (HBSS) and 10% FBS and triturated mechanically. The viability and number of neuronal cells were calculated automatically in an Automated Cell Counter (Bio-Rad), plated at a density of 1,000,000 cells per well on 6-well plates and cultured in Neurobasal A Medium (Thermo Fisher Scientific (Gibco), #12349015, Waltham, MA, USA) supplemented with GlutaMAX (Thermo Fisher Scientific (Gibco) #A1286001, Waltham, MA, USA), B-27 Supplement (Thermo Fisher Scientific (Gibco), #17504001, Waltham, MA, USA), and 200 ng/mL Primocin (Invitrogen, ant-pm-1, San Diego, CA, USA).

#### Proteasome Activator Treatments

Mice primary cortical neurons were treated with **1g** and **1f** proteasome activators at a concentration of 2.5 μM/ml at day 7 in vitro (DIV7) and incubated for 7 days. On day 14 (DIV14), cell lysates were collected for further analysis.

#### Protein Extraction and Quantification

Mice primary cortical neurons were washed twice with PBS and lysed in HTRF P-T prot.—Lysis Buffer 5 (PerkinElmer, #64KL5FDF, Waltham, MA, USA) with Halt Phosphatase Inhibitor Cocktail (Thermo Fisher Scientific, #78420, Waltham, MA, USA) and cOmplete Mini Protease Inhibitor Cocktail (Merck, #11836153001, DA, Germany). Cell lysates were collected on ice, centrifuged at 16,000*g* for 15 min, and supernatants were frozen at −20 °C. Protein concentrations in the cell lysates were determined using a bicinchoninic acid (BCA) assay (Sigma-Aldrich, 3B9643-1L-KC, MO, USA) according to the manufacturer’s instructions.

#### Simple Western Jess Analysis

To measure abundance of α-synuclein, we used anti-α-synuclein antibody (BD Biosciences, #610786, NJ, USA) together with the Simple Western Jess (Bio-Techne, ProteinSimple, MN, USA) automated Western blot, utilizing the Fluorescence Separation Module 12-230 kDa (Bio-Techne, #SM-FL004, MN, USA) and the Protein Normalization Module (Bio-Techne, #AM-PN01, MN, USA) according to manufacturer’s instructions, and using 0.13 to 0.5 mg of total protein. For the signal detection, the chemiluminescence method was performed, captured, and quantified using Compass for SW Software (Compass for SW, ProteinSimple, MN, USA). The relative protein levels were normalized to the total protein signal for each capillary (Corr. Area). Statistical analysis was conducted in GraphPad Prism (version 10.1.1, GraphPad Software, CA, USA), with one-way RM-ANOVA and Fisher’s multiple comparison test.

#### Studies with α-Synuclein Aggregation in a Mouse Primary Cell Model of Parkinson’s Disease

Cells were isolated from pregnant mouse embryos at E16.0 and plated on precoated with poly-l-lysine wells of 96-well plates (PerkinElmer/Revvity Inc., Waltham, MA, USA) at 25,000 cells/well. The final medium volume was 150 μL/well. Cells were incubated at 37 °C, 5% CO_2_. The medium consisted of NB medium (Neurobasal Medium [−] l-Glutamine, Gibco, #21103-049), B27 neuronal cell culture supplement (2%) (Gibco/Thermo Fisher Scientific, #17504044, Waltham, MA, USA), l-glutamine (0.25%) (Gibco, #25030-032), and Primocin (0.2%) (InvovoGen, #ant-pm-1, San Diego, CA, USA). The medium was partially replaced on the third (−25 μL/+75 μL) and on the seventh (−75 μL/+75 μL) day of the experiment.

#### Induction of α-Synuclein Aggregates in Cells and Introduction of Modulators into the Cell Culture

The solution of active mouse recombinant alpha synuclein protein preformed fibrils (PFFs, StressMarq, #SPR-324B) was diluted with PBS pH 7.4 (1×; Gibco, #10010-031) to a concentration of 100 μg/mL and sonicated (10 cycles of 30 s ON/30 s OFF, 4 °C). On the eighth day of the experiment, 3.75 μL of PFF solution/well was added (the final PFF concentration in a well was 2.5 μg/mL). The control wells were treated with the same volume of PBS (1×). After 1 h of incubation at 37 °C, 5% CO_2_, 1.5 μL of the tested modulators at a concentration of 0.5 and 2.5 μM was added to the cells with PFFs. For the control samples, the same volume of PBS was added.

#### Immunostaining

On day 15 of the experiment, the medium was withdrawn and 4% paraformaldehyde (PFA) was added in an amount of 50 μL/well. The plate was incubated for 20 min at room temperature. After cell fixation, PFA was removed, and all wells were washed three times with PBS solution. In the next step, permeabilization was performed: PBS was removed, and the cells were incubated with a 0.2% Triton X-100 solution in PBS (PBST) for 15 min at room temperature. The PBST solution was withdrawn from the wells, and then, the cells were incubated for an hour with a 5% normal horse serum (NHS) solution in PBST at room temperature. A solution of primary antibodies against pS129-αsyn (Rb@pαsyn, 1:2000) (Abcam, Cambridge, UK) and neuron-specific protein NeuN (M@NeuN 1:500) (Abcam, Cambridge, UK) was prepared in a 5% NHS/PBST solution. After incubation of the cells with 5% NHS/PBST, the solution was gently removed and 40 μL of the primary antibody solution/well was added and incubated for 3 h at room temperature. After this time, the antibodies were removed, and all wells were washed three times with the PBS solution. In the next step, fluorescently labeled secondary antibodies diluted in PBST were added to the cells: D@M647 (1:500) (Thermo Fisher Scientific, Waltham, MA, USA) and D@R488 (1:500) (Thermo Fisher Scientific, Waltham, MA, USA), 40 μL/well. After incubation for 1 h at room temperature, the contents of the wells were aspirated and the cells were washed three times with PBS. The last step was DAPI staining. For this purpose, a 1 mg/mL dye solution (Thermo Fisher Scientific, Waltham, MA, USA) was diluted in a ratio of 1:5000 with PBS. 40 μL/well DAPI solution was added and incubated for 10 min. Finally, the cells were again washed three times with PBS. The plate was scanned using an ImageXpress Nano Automated Imaging System microscope (Molecular Devices, San Jose, CA, USA) with three fluorescent filters. The acquired images were analyzed using the CellProfiler and CellProfiler Analyst software packages.^37^

#### Microscale Thermophoresis (MST) Assay

The 20S proteasome was labeled following the manufacturer’s protocol using the RED-NHS 2nd Generation labeling kit, NT-647 (NanoTemper Technologies GmbH, Munich, Germany). Monolith X (NanoTemper) was used to study the interaction between the fluorescently labeled enzyme and the activators. The assay buffer was 50 mM Tris/HCl, pH 7.2, supplemented with 5% glycerol. Serial dilutions of the activators, spanning 16 concentrations from 1.5 nM to 50 μM, were prepared and mixed with the labeled proteasome, whose concentration was kept constant at 10 nM. The IR laser radiation intensity was automatically optimized by the system. MST data were analyzed by fitting the resulting curves using the Hill equation in OriginPro 2021. EC_50_ values were determined from the results of three independent experiments.

## Supporting Information Available

Effect of unnatural amino acids on stability of modulators; ChT-L, T-L, and C-L activity of the human 20S proteasome in the presence of the modulators; SDS PAGE gels of protein degradation by h20S in the presence of activators; and HPLC and HR MS of the activators (CSV)
Molecular formula strings with potency data (PDF)

## Abbreviations

AD: Alzheimer’s disease
AMC: aminomethylcoumarin
C-L: caspase-like activity
ChT-L: chymotrypsin-like activity
CP: core particle
Dabcyl-EDANS: Lys(Dabcyl)-Met-Ser-Gly-Phe-Ala-Ala-Thr-Ala-Glu(EDANS)-Gly FRET-type proteasome substrate
HbYX: a moiety consisting of Hb-hydrophobic, Y-tyrosine, and X-any amino acid
PD: Parkinson’s disease
SEM: standard error of the mean
T-L: trypsin-like activity
