# Supplementary material for: Peptidomimetics Activating the Proteasome: A New Perspective for Parkinson’s Treatment
Source: J Med Chem. 2025 Apr 7;68(8):8967–79. doi: 10.1021/acs.jmedchem.5c00645 (PMC12035797; doi:10.1021/acs.jmedchem.5c00645)

Supporting Information

Peptidomimetics Activating the Proteasome: A New Perspective for Parkinson's Treatment

Karolina Trepczyk [a], Safak Er [b], Irena Hlushchuk [b], Mikko Airavaara [b], Anna Alwani [c], Katarzyna Maziarz [c], Piotr Chmielarz [c], Kinga Słomska [a], Ewa Wieczerzak [a]*, Elżbieta Jankowska [a]*

[a] Department of Biomedical Chemistry, Faculty of Chemistry, University of Gdansk, Wita Stwosza 63, 80-308 Gdańsk, Poland

[b] Pharmacology and Drug Development Division of Pharmacology and Pharmacotherapy, Faculty of Pharmacy, FI-00014 University of Helsinki, Helsinki, Finland

[c] Department of Brain Biochemistry, Maj Institute of Pharmacology, Polish Academy of Sciences, Smętna 12, 31-343 Kraków, Poland

E-mail: elzbieta.jankowska@ug.edu.pl

E-mail: ewa.wieczerzak@ug.edu.pl

Contents of SI:

- Table illustrating the weak effect of unnatural amino acids on improving the stability of modulators (S2)
- Figures displaying the activity of human 20S proteasome in the presence of the modulators, probed using Suc-LLVY-AMC, Boc-LRR-AMC and Z-LLE-AMC substrates (S3-S4)
- Figures displaying the ChT-L and T-L activity of human 20S proteasome in the presence of the modulators with the CPP sequence attached (S5)
- SDS PAGE gels of α-synuclein and Tau-441 degradation by h20S in the presence of activators (S6)
- HPLC and HR MS analysis of the designed analogues (S7-S19)

Table S1. Results of digestion of Blm analogs by the h20S proteasome. Digestion sites in Blm peptides and peptidomimetics were identified by LC-MS.

Name	Sequence	% undigested peptide
		1 h
**3**	KYFTGSKEWRSYYT	8
**3a**	KYFTGSKEW-^{nitro}R-SYYT	23
**3b**	K-$^{4\text{-}fluoro}$F-FTGSKEWRS-$^{4\text{-}fluoro}$F- YT	7
**4**	KYFTGSKYDRRYYS	58
**4a**	KYFTGSKYD-^{nitro}R- ^{nitro}R-YYS	30
**4b**	K-$^{4\text{-}fluoro}$F-FTGSK-$^{4\text{-}fluoro}$F-DRR-$^{4\text{-}fluoro}$F-YS	83[1]
**5**	KFTQKPLWRSYYA	3
**5a**	KFTQKPLW- ^{nitro}R-SYYA	16
**5b**	KFTQKPLWRS-$^{4\text{-}fluoro}$F-YA	2

[1] analog **4b** did not have the ability to activate h20S

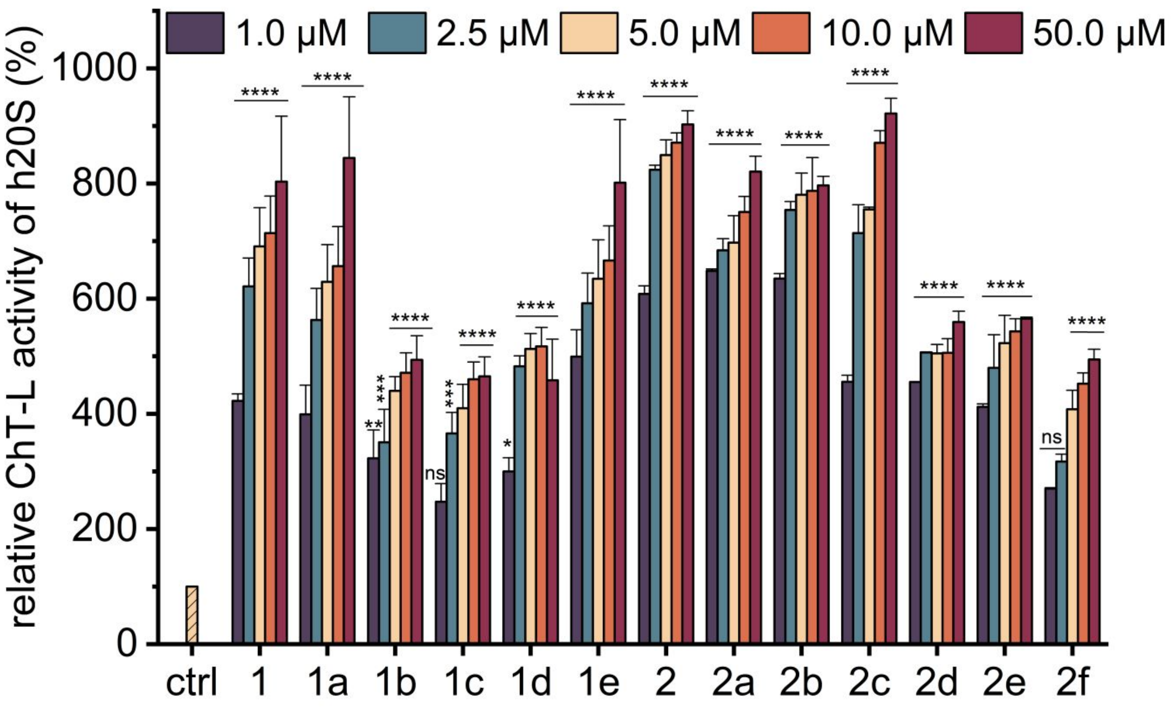

Figure S1. Influence of Blm modulators on the activity of human 20S proteasome, probed with Suc-LLVY-AMC substrate. The results are presented as the mean ± SEM.

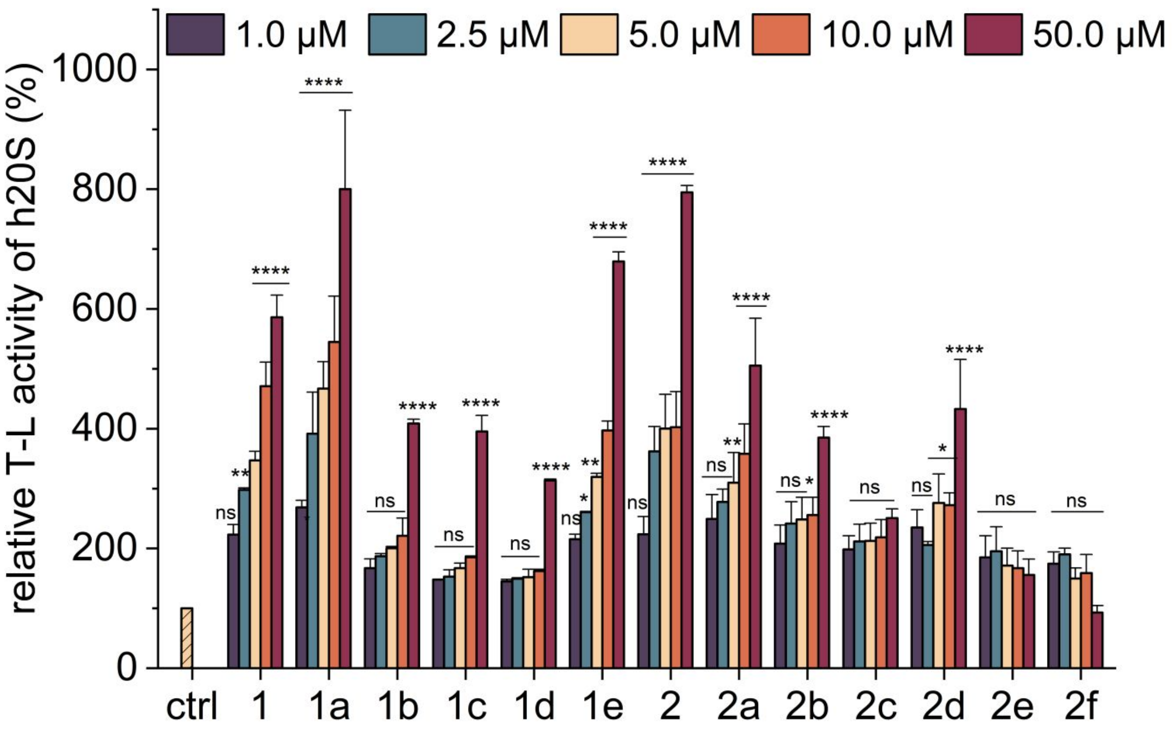

Figure S2. Influence of Blm modulators on the activity of human 20S proteasome, probed with Boc-LRR-AMC substrate. The results are presented as the mean ± SEM.

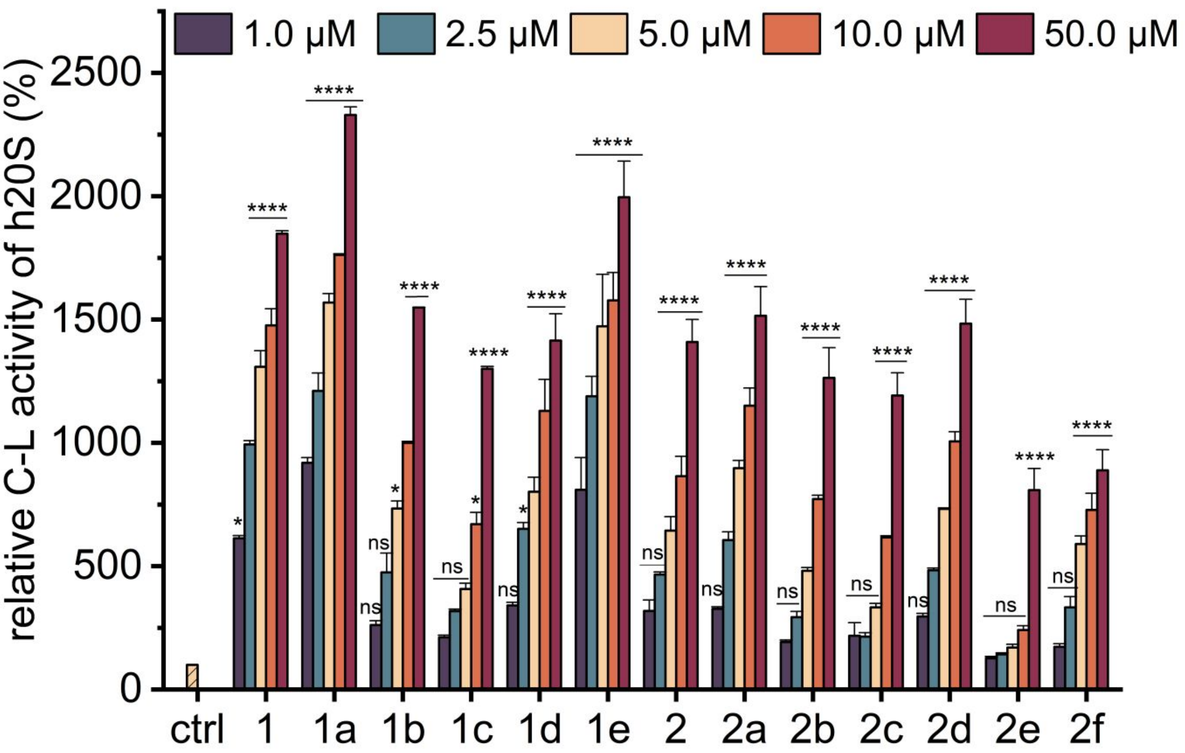

Figure S3. Influence of Blm modulators on the activity of human 20S proteasome, probed with Z-LLE-AMC substrate. The results are presented as the mean ± SEM.

a) b)

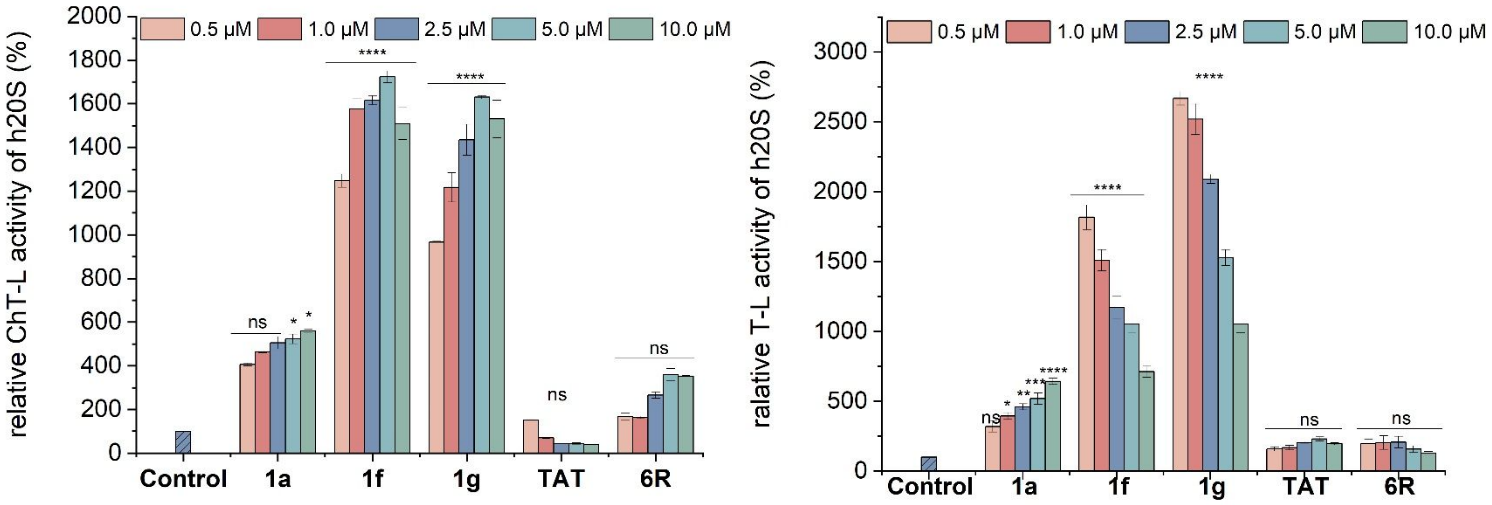

Figure S4. Influence of Blm-CPP modulators on the chymotrypsin-like (a) and trypsin-like (b) activities of the human 20S proteasome, probed with 100 μM Suc-LLVY-AMC and Boc-LRR-AMC substrates, respectively. The results are presented as the mean ± SEM.

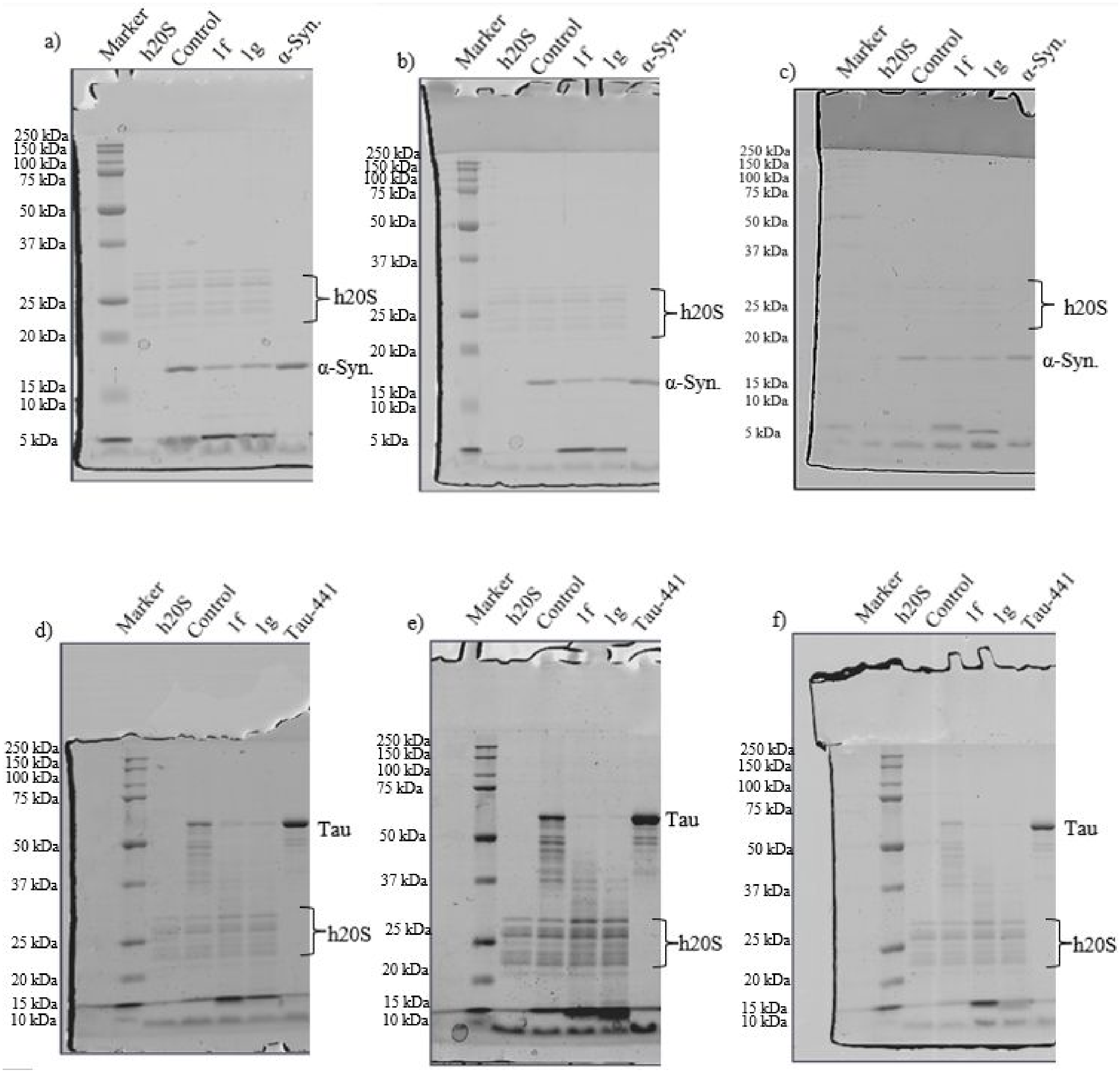

Figure S5. Protein degradation by h20S in the absence and presence of Blm modulators. SDS PAGE gels of α-synuclein (a, b, c) and Tau-441 (d, e, f) degradation by h20S in the presence of activators at 10 μM concentration. Precision Plus Protein™ Dual Xtra Prestained Protein Standards (Biorad) was used as a marker.

Identity and purity of the activators confirmed by MS spectra and HPLC analysis, respectively:

1a

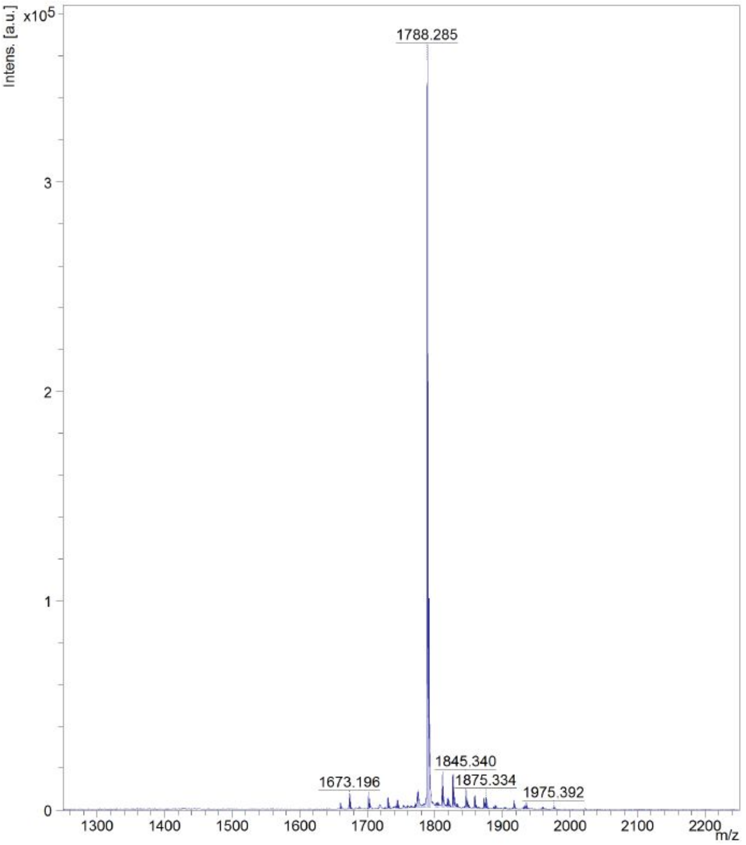

calculated:

MW 1787.519

$[M+1H]^{+1}$ 1788.285

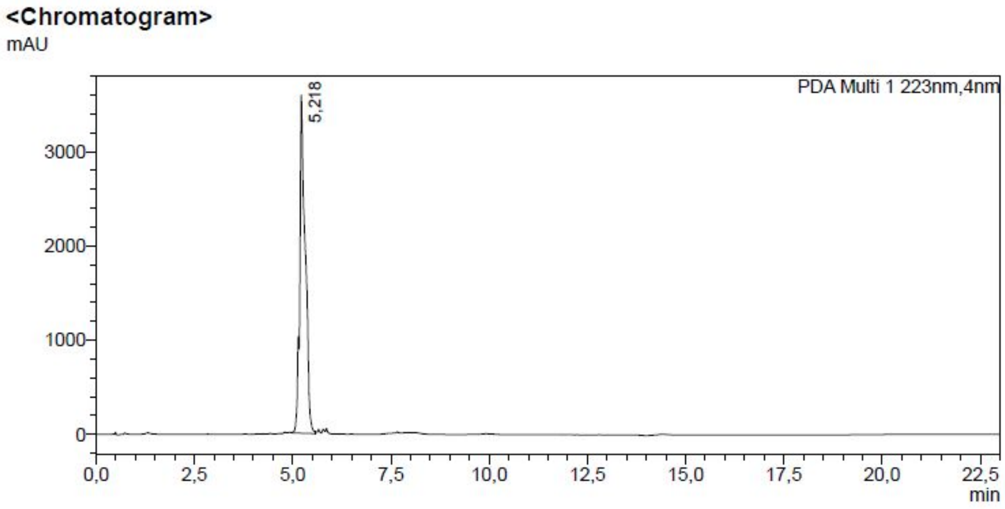

1b

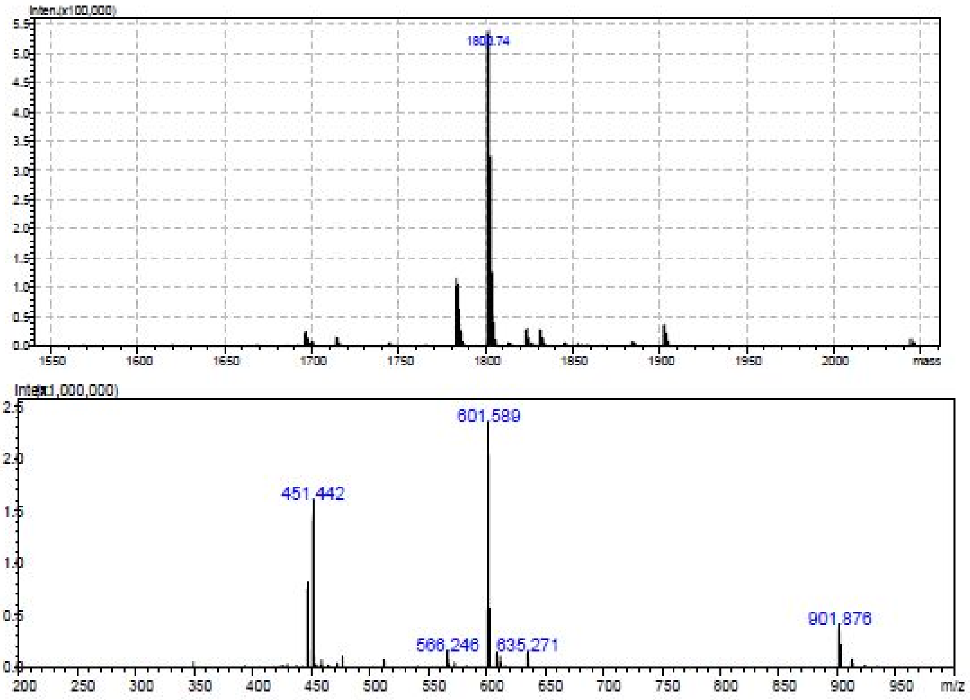

calculated:

MW 1800.745

$[M+2H]^{+2}$ 901.876

$[M+3H]^{+3}$ 601.589

$[M+4H]^{+4}$ 451.442

<Chromatogram>

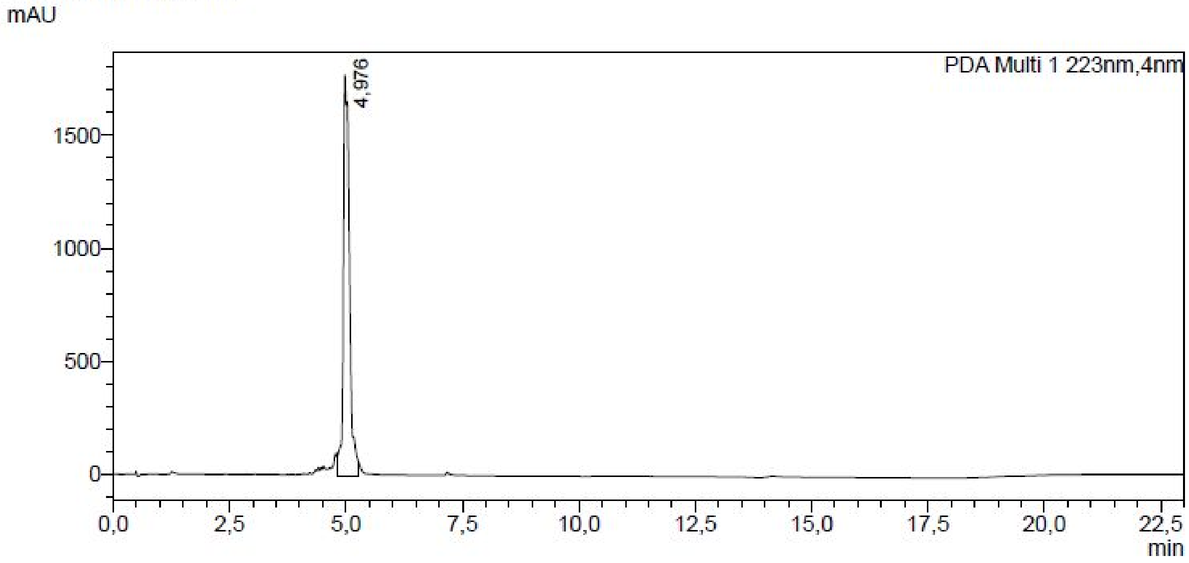

1c

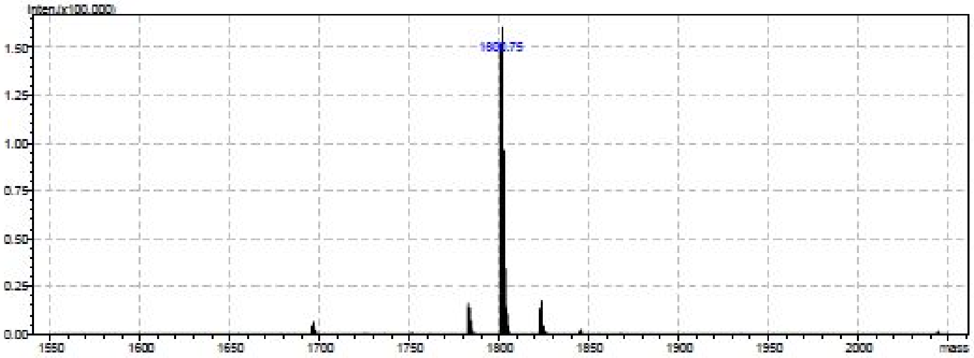

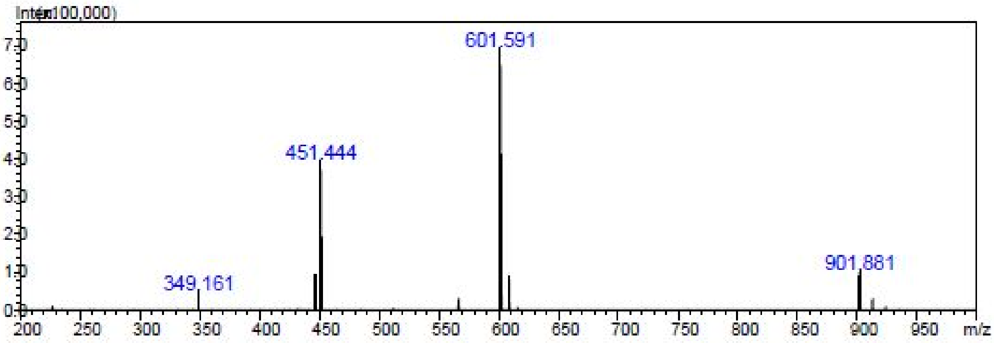

calculated:

MW 1800.745

$[M+2H]^{+2}$ 901.881

$[M+3H]^{+3}$ 601.591

$[M+4H]^{+4}$ 451.444

<Chromatogram>

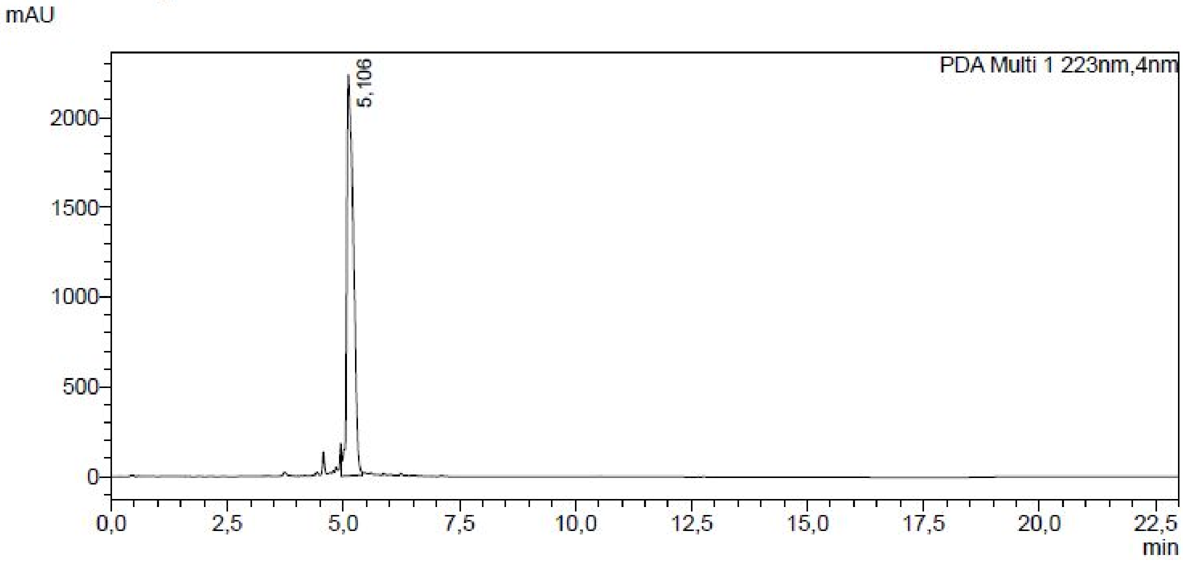

1d

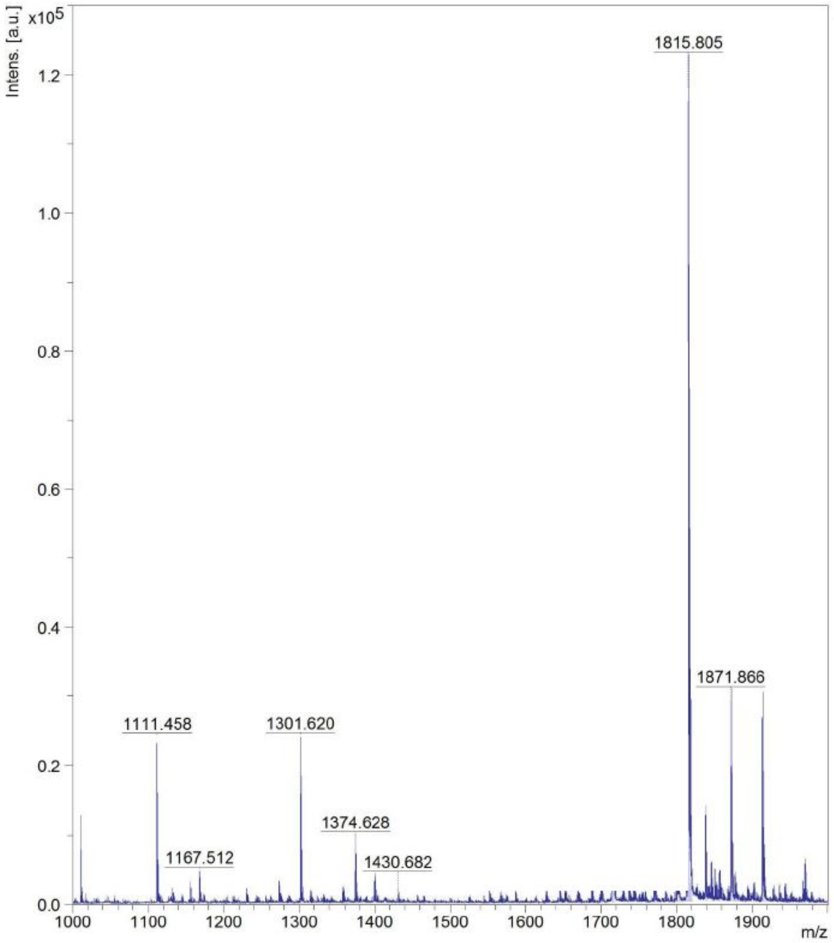

calculated:

MW 1814.806

$[M+1H]^{+1}$ 1815.805

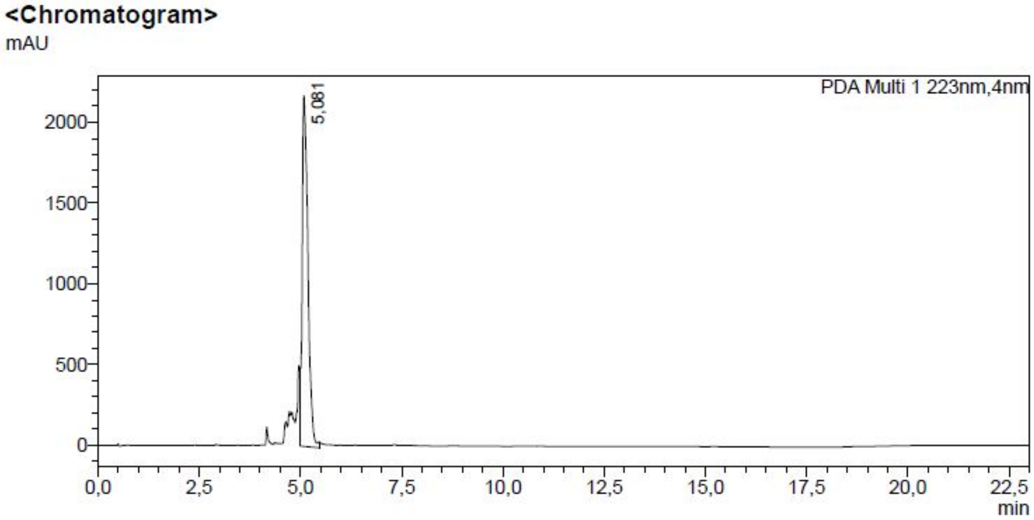

1e

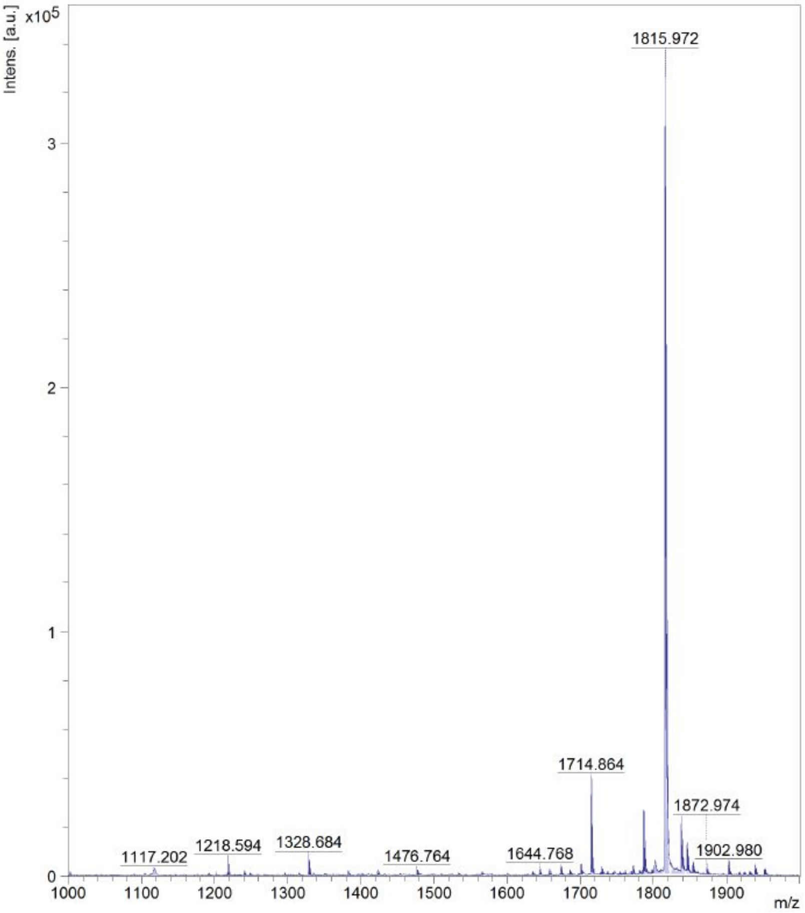

calculated:

MW 1814.806

$[M+1H]^{+1}$ 1815.972

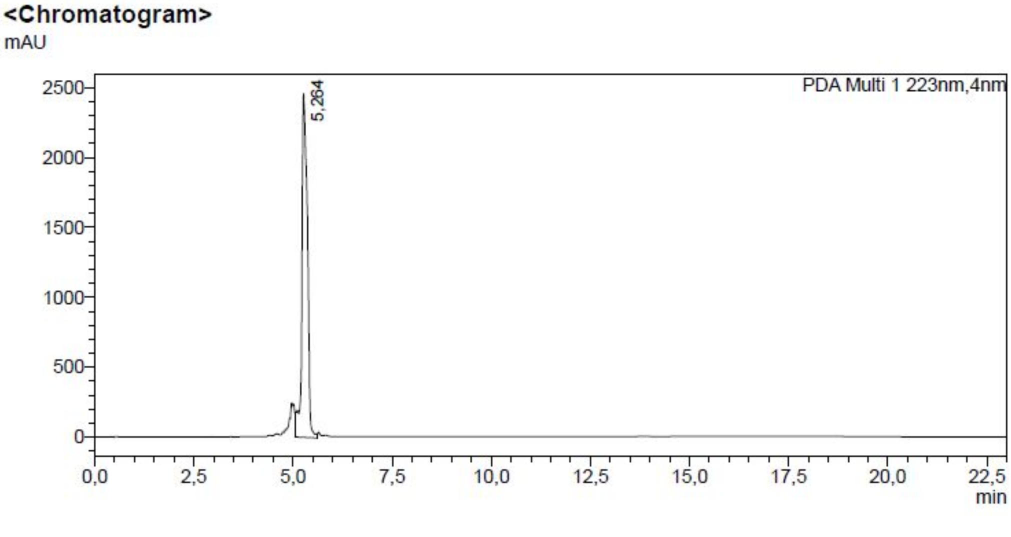

1f

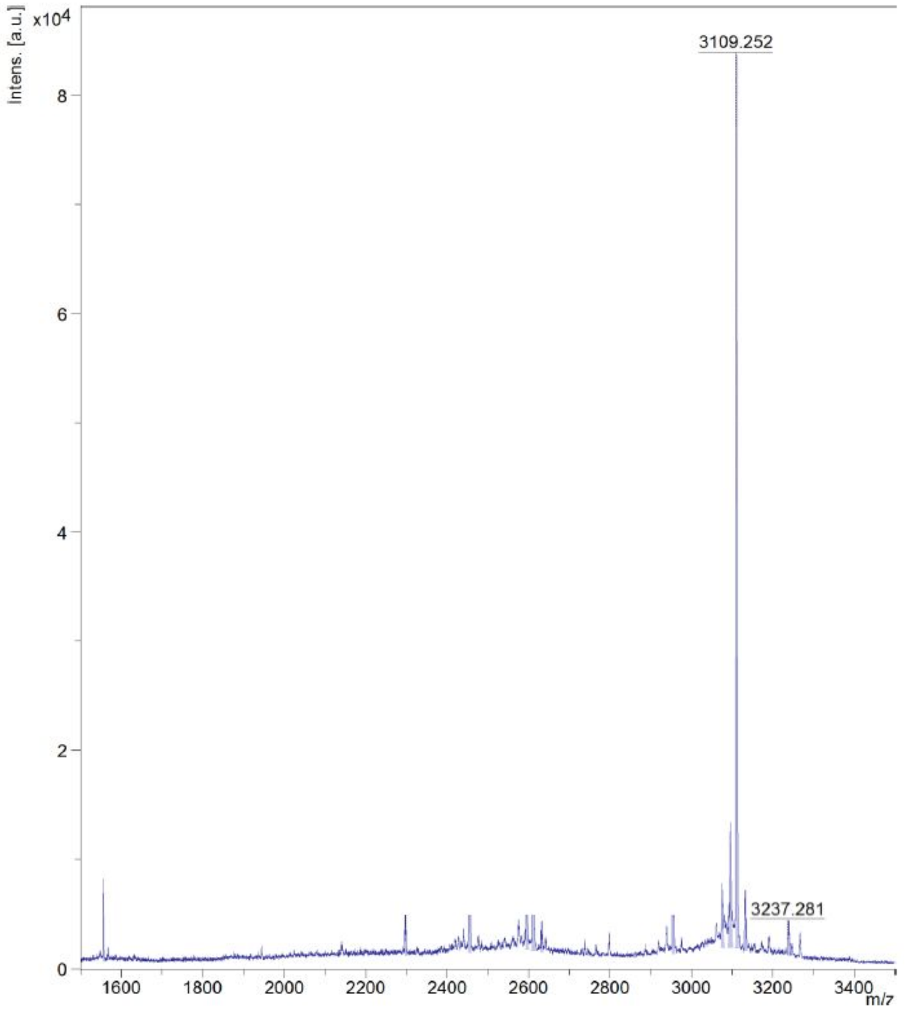

calculated:

MW 3108.529

$[M+1H]^{+1}$ 3109.252

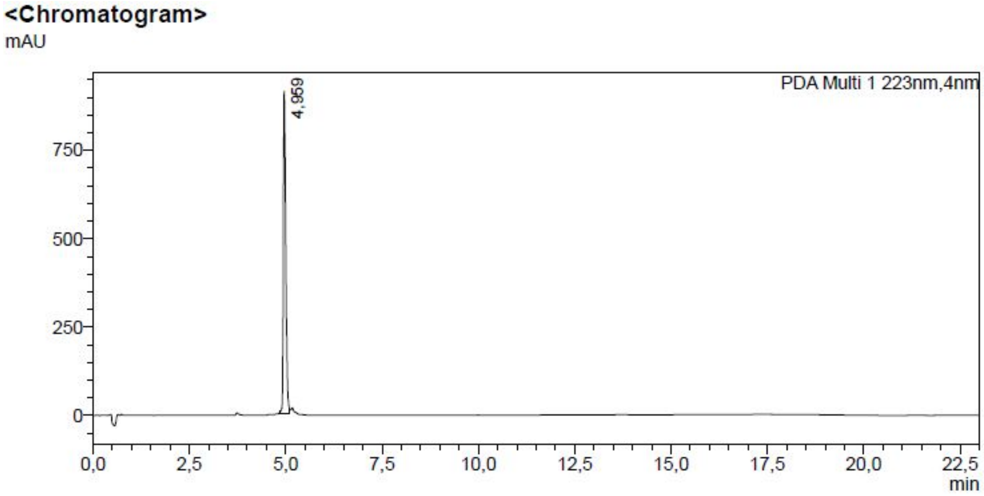

1g

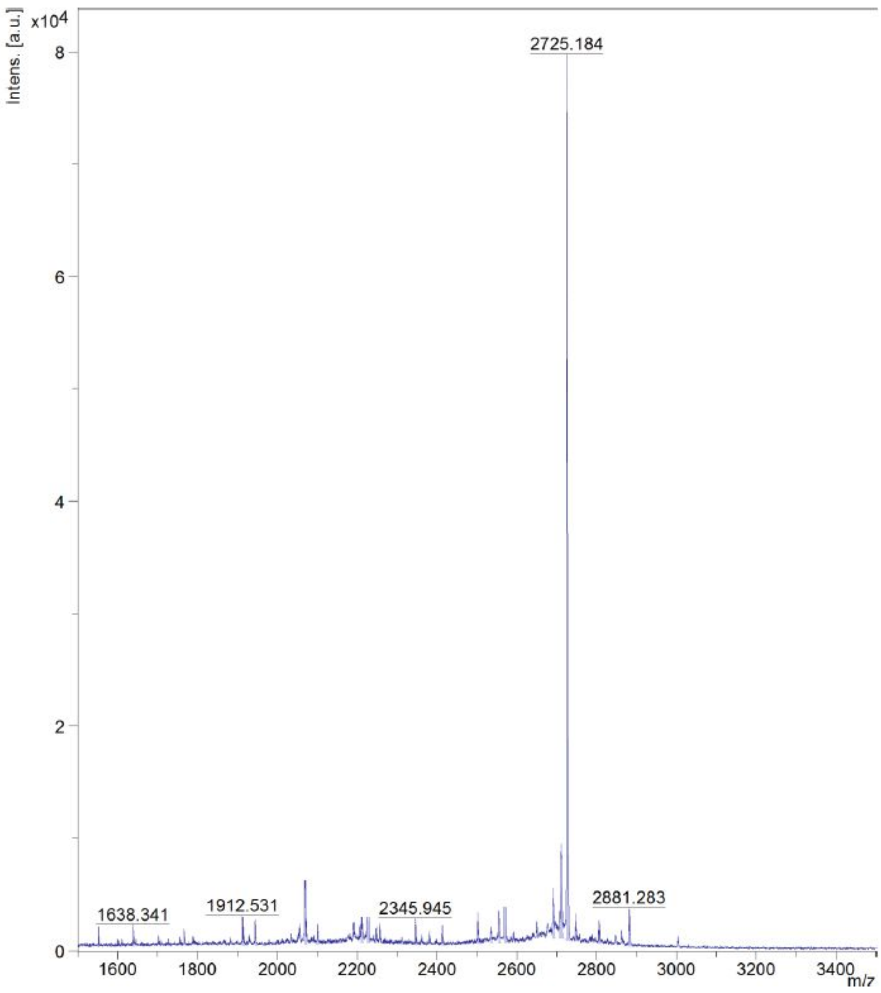

calculated:

MW 2724.051

$[M+1H]^{+1}$ 2725.184

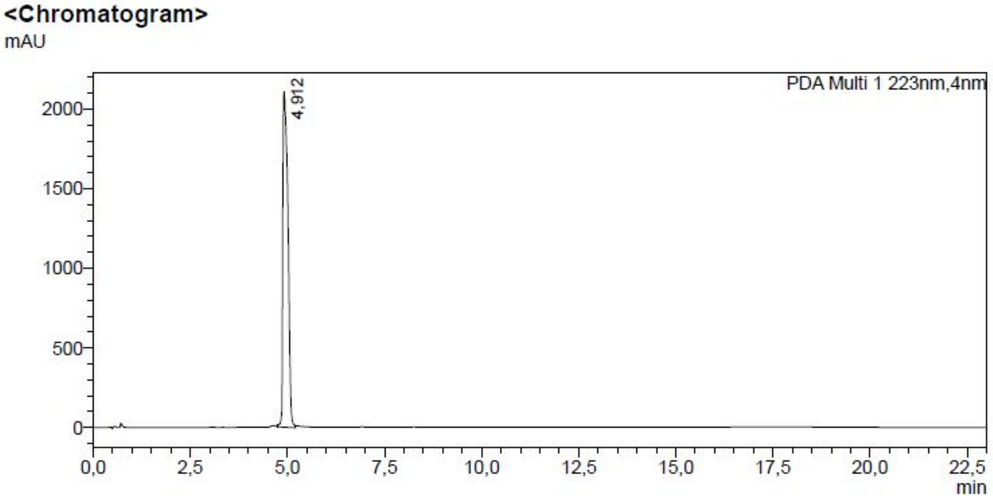

2a

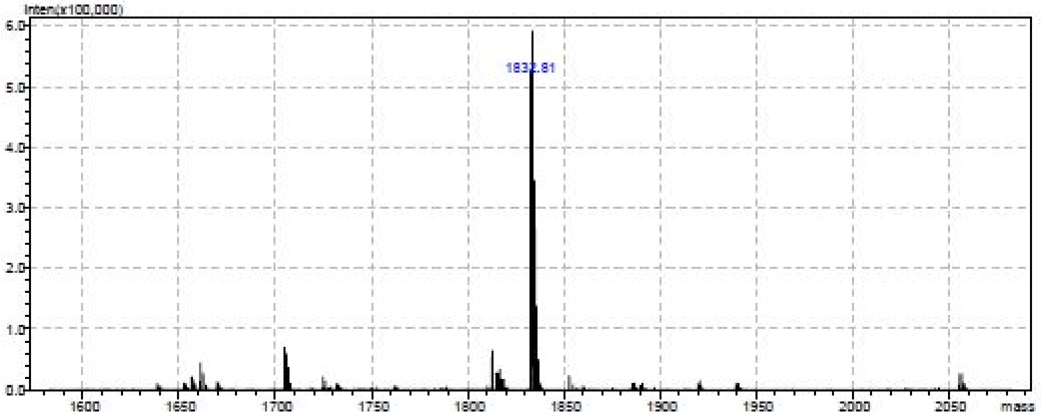

calculated:

MW 1832.811

$[M+3H]^{+3}$ 612.281

$[M+4H]^{+4}$ 459.461

$[M+5H]^{+5}$ 364.166

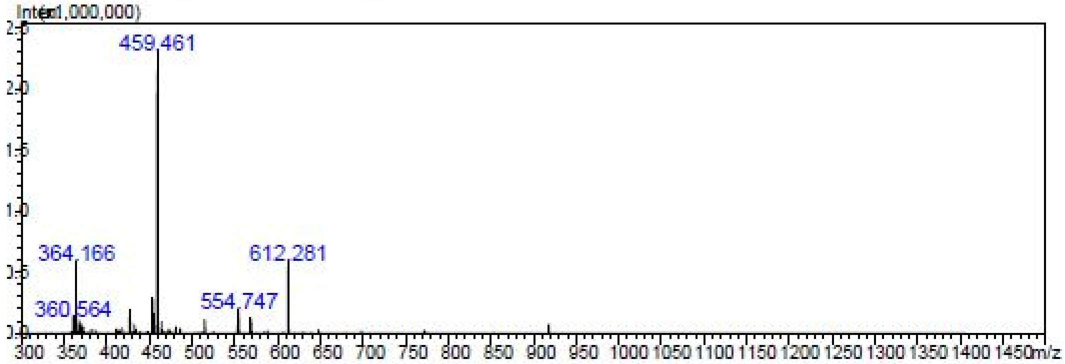

<Chromatogram>

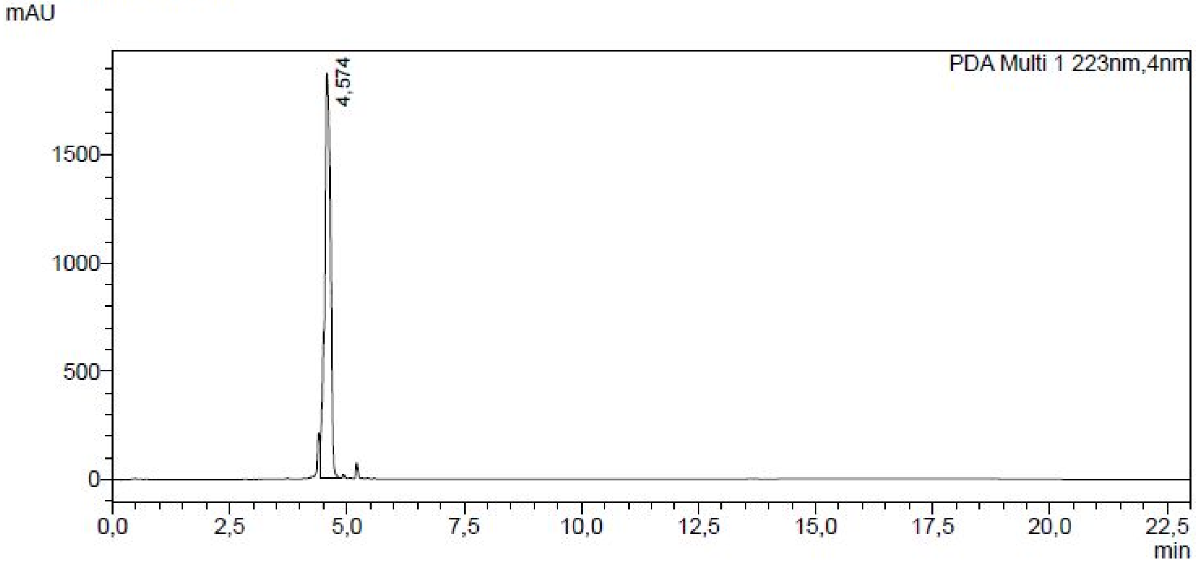

2b

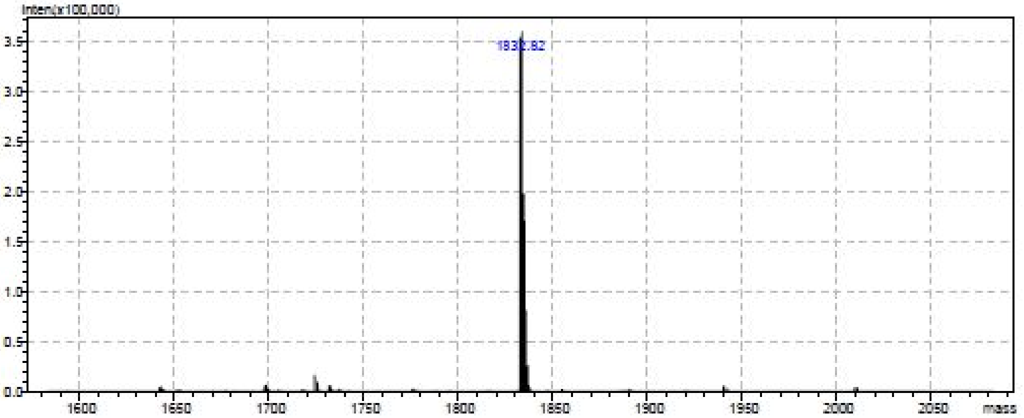

calculated:

MW 1832.823

$[M+2H]^{+2}$ 917.914

$[M+3H]^{+3}$ 612.281

$[M+4H]^{+4}$ 459.460

$[M+5H]^{+5}$ 364.168

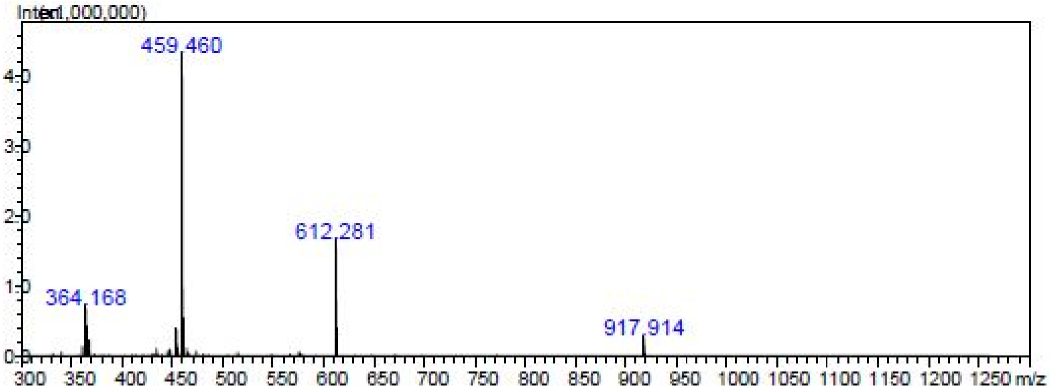

<Chromatogram>

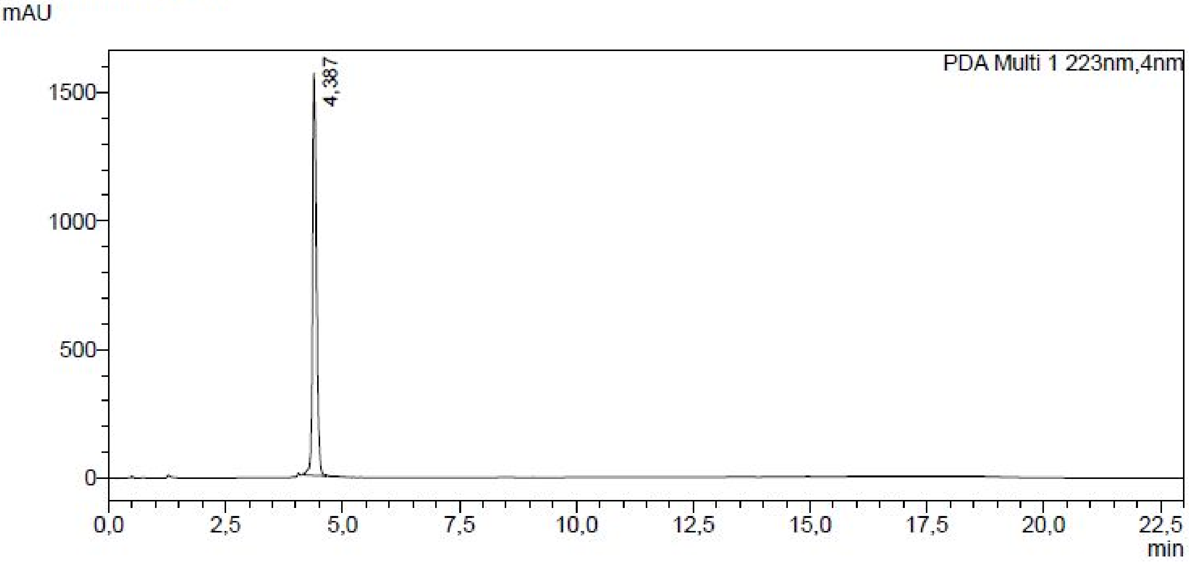

2c

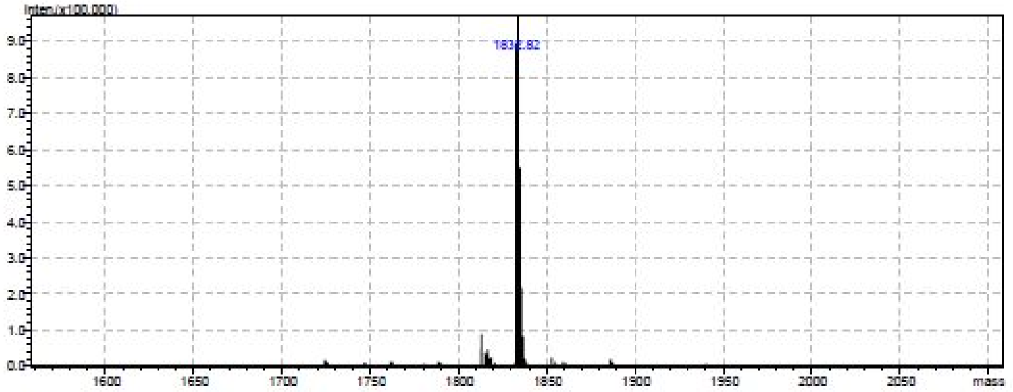

calculated:

MW 1832.823

$[M+3H]^{+3}$ 612.282

$[M+4H]^{+4}$ 459.461

$[M+5H]^{+5}$ 364.169

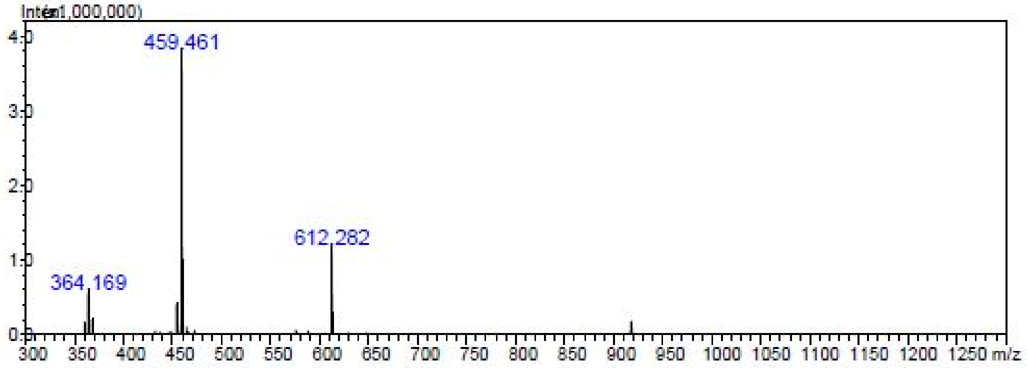

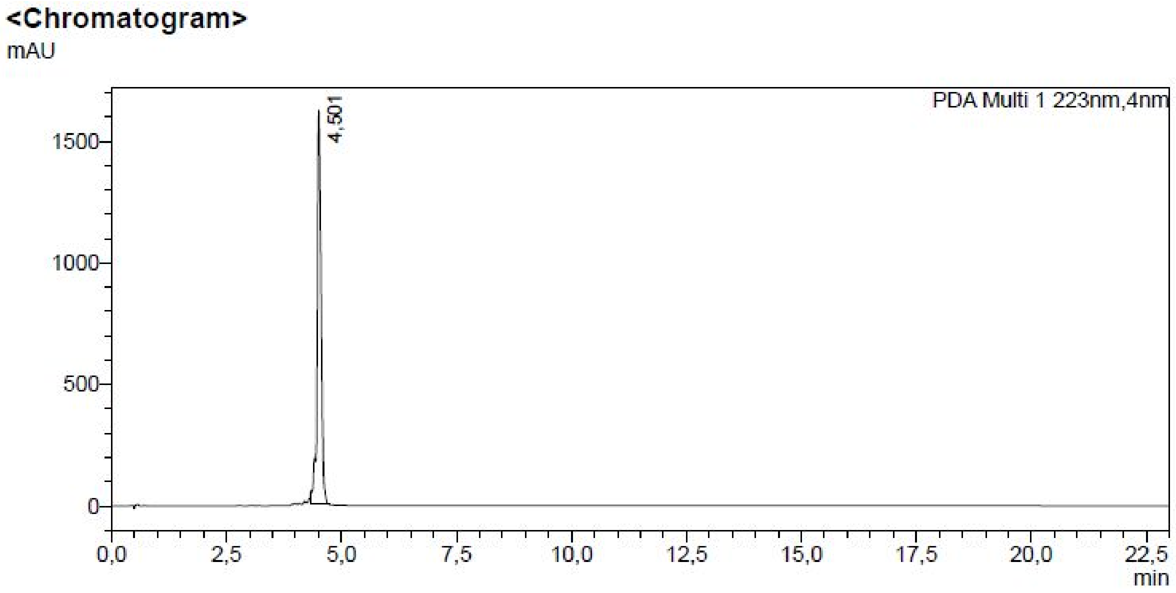

2d

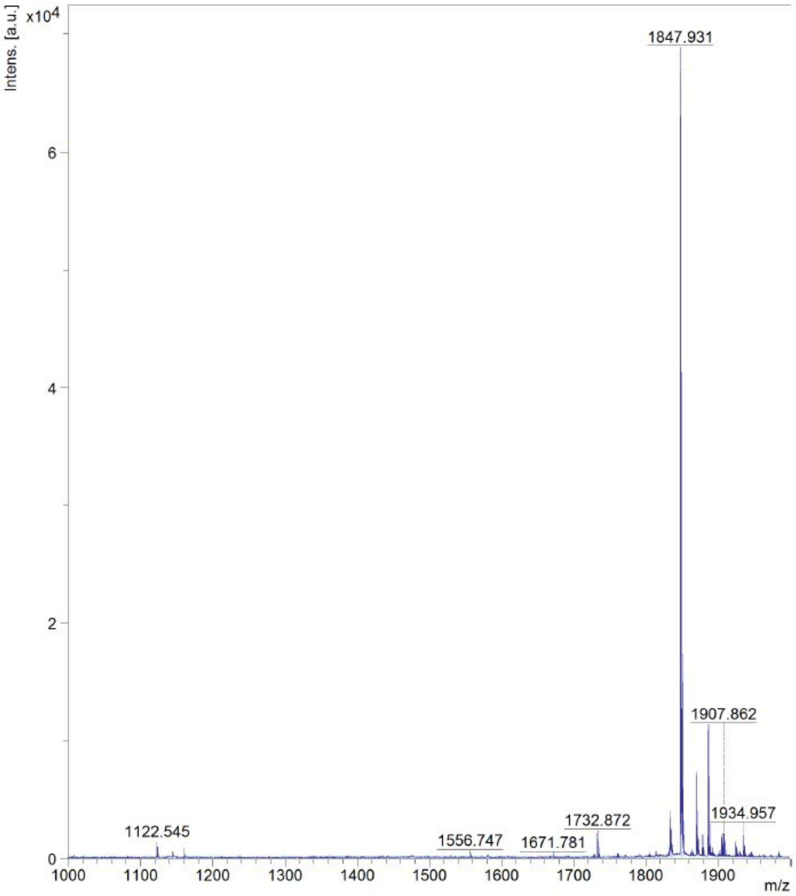

calculated:

MW 1846.621

$[M+1H]^{+1}$ 1847.931

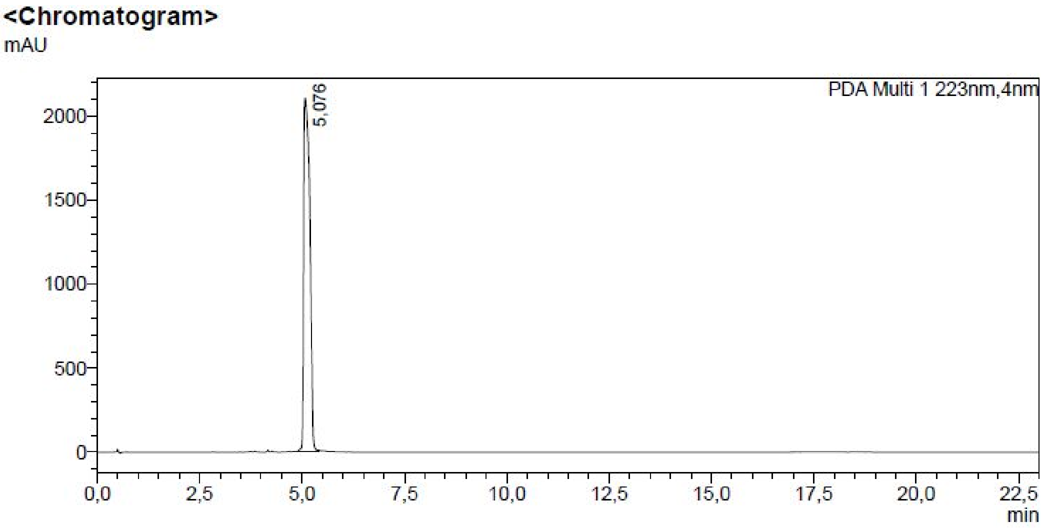

2e

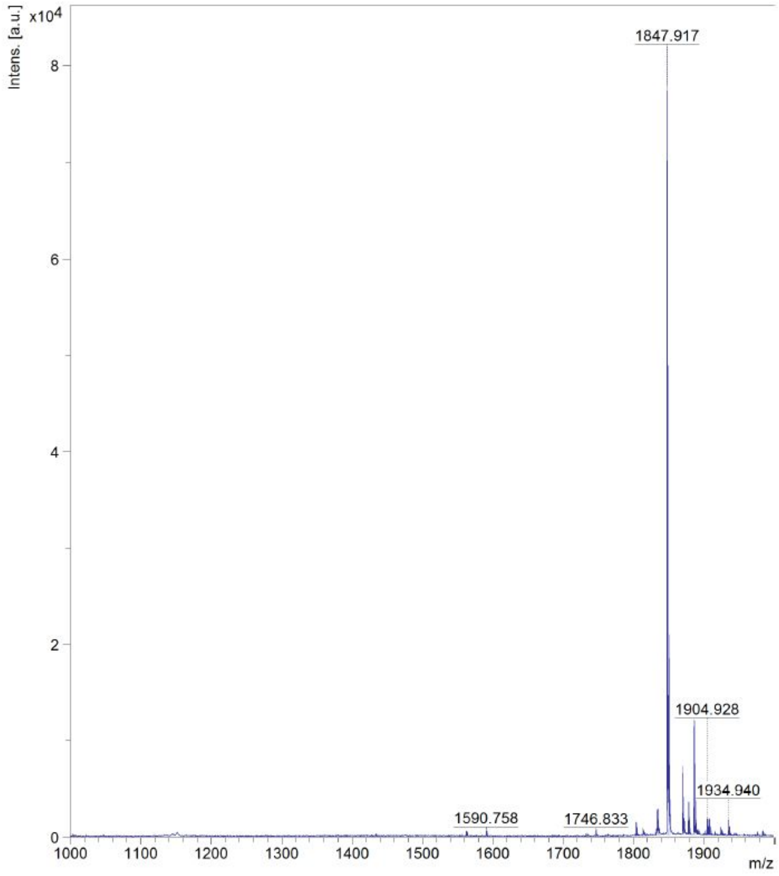

calculated:

MW 1846.621

$[M+1H]^{+1}$ 1847.971

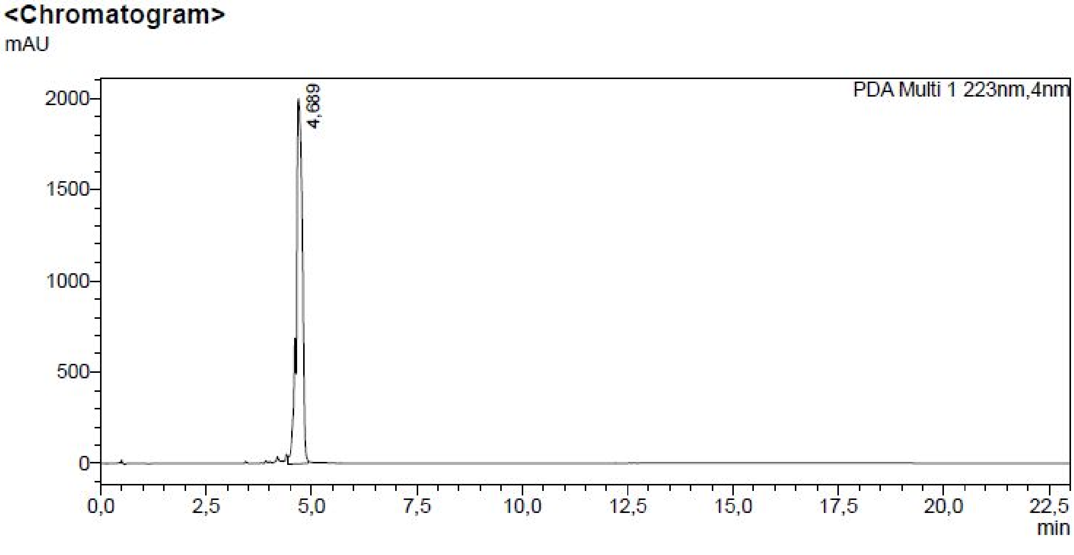

2f

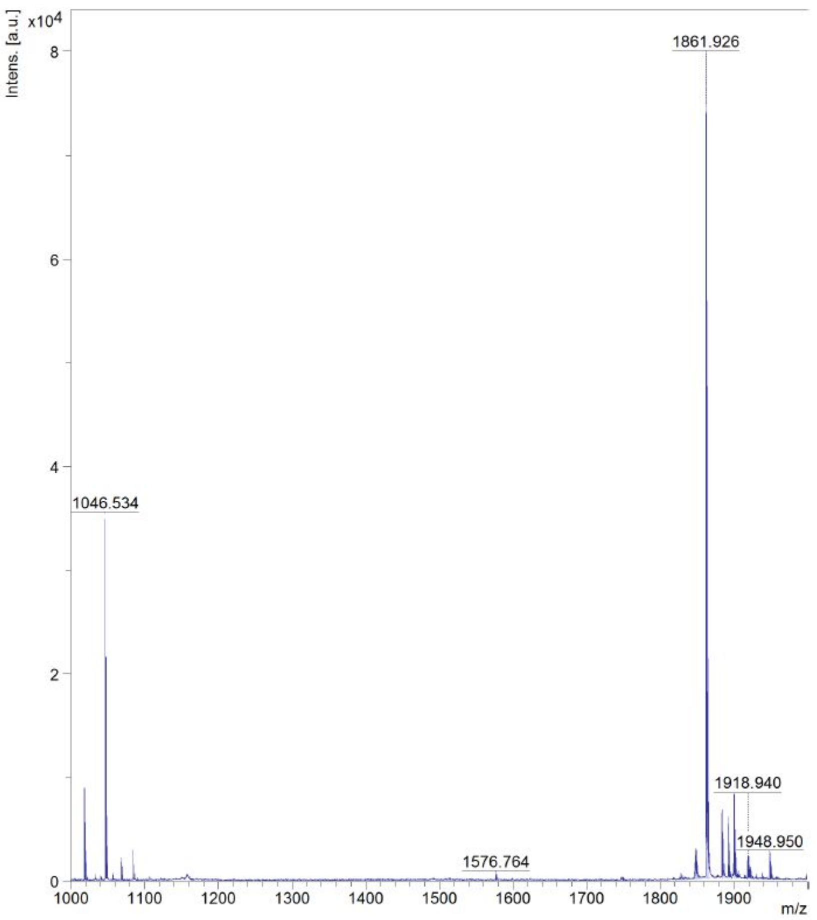

calculated:

MW 1860.799

$[M+1H]^{+1}$ 1861.926

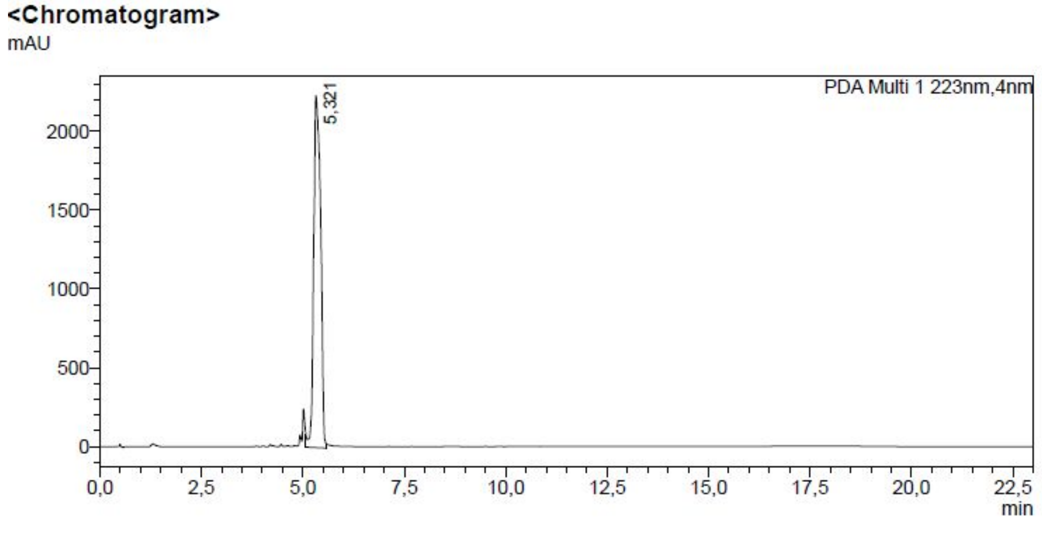

Supplement: Supplementary file 2 — jm5c00645_si_002.pdf [file jm5c00645_si_002.pdf]
